# *Amomum tsao-ko Crevost et Lemarie* extract targets the gut-liver axis to combat atherosclerosis in ApoE^−/−^ mice

**DOI:** 10.3389/fmicb.2025.1641035

**Published:** 2025-10-31

**Authors:** Qianqian Wang, Yuanyuan Niu, Jiawei Huang, Junhong Huang, Jiamin Zhang, Boyi Zhang, Zixuan Guo, Shuying Feng

**Affiliations:** ^1^Medical College, Henan University of Chinese Medicine, Zhengzhou, China; ^2^Henan Engineering Research Center for Chinese Medicine Foods for Special Medical Purpose, Zhengzhou, China

**Keywords:** atherosclerosis, gut microbiota, oxidative stress, ApoE^−/−^ mice, hepatic inflammation, 16S rRNA sequencing

## Abstract

**Background:**

*Amomum tsao-ko Crevost et Lemarie* (T-K), a valuable dual-purpose plant used in both medicine and food, exhibits a wide array of bioactivities and pharmacological effects, including the regulation of gastrointestinal function, promotion of weight loss and fat reduction, lowering of blood sugar levels, antioxidant activity. The efficacy of T-K and its underlying mechanism in managing atherosclerosis have rarely been discussed in the literature. This research aimed to evaluate the therapeutic potential of T-K in atherosclerotic mouse models induced by a high-fat high-cholesterol (HFHC) diet and to explore the potential mechanisms involved.

**Methods:**

Atherosclerotic mice were fed with an HFHC diet for 12 weeks, followed by a continuous oral administration of T-K extract via gavage for an additional 8 weeks. Full-length aorta Oil Red O staining, aortic root Oil Red O staining, and hematoxylin–eosin staining of liver tissues were employed to assess the efficacy of T-K. Biochemical methods and enzyme-linked immunosorbent assays were utilized to quantify alterations in inflammatory markers and oxidative stress indicators in serum and liver tissues. 16S rRNA sequencing technology was used to analyze alterations in the composition of the intestinal microbiota in animals following treatment with T-K.

**Results:**

Full-length aorta Oil Red O staining, aortic root Oil Red O staining, and liver hematoxylin–eosin staining effectively evaluated the therapeutic potential of T-K in managing atherosclerosis. Serological tests confirmed T-K’s ability to decrease the total serum cholesterol and low-density lipoprotein cholesterol levels. Additionally, gut microbiota showed significant alterations following T-K treatment, which were markedly different from the changes observed after statin therapies. Furthermore, the results from the enzyme-linked immunosorbent assay indicated that T-K significantly reduced inflammation in both the aorta and liver. Oxidative stress assessments revealed that T-K can mitigate oxidative stress and thus improve atherosclerosis.

**Conclusion:**

T-K has demonstrated significant efficacy in the treatment of atherosclerosis, primarily by lowering serum cholesterol levels and modulating intestinal flora at multiple levels to enhance disease management. Moreover, T-K mitigated the disease progression by attenuating oxidative stress and inflammatory responses in both the liver and aorta.

## 1 Introduction

Cardiovascular diseases (CVDs) continue to be the leading cause of death worldwide, significantly impacting public health and contributing to excessive healthcare costs. The global incidence of CVDs increased from 271 million (12.1 million death) in 1990 to 523 million (18.6 million death) in 2019, nearly doubling during this period (Roth et al., 2020; Vaduganathan et al., 2022). Atherosclerosis is a major contributor to CVDs globally (Herrington et al., 2016). It is regarded as a multifactorial metabolic condition, with key risk factors comprising hypertension, elevated lipid levels, smoking, diabetes, obesity, and genetic susceptibility (Falk, 2006). The mechanisms underlying atherosclerosis involve multiple factors, such as high cholesterol, inflammation, endothelial dysfunction, immune response, smooth muscle cell dysfunction, and plaque rupture (Libby et al., 2019). In addition, recent studies have increasingly emphasized the role of intestinal microbes in the onset and progression of atherosclerosis (Björkegren and Lusis, 2022). Although pathogenesis of atherosclerosis is complex, multiple treatments are available, including statins that regulate lipid metabolism, antiplatelet drugs, medications that improve endothelial function, and procedures such as stent implantation, interventional surgery, and endarterectomy (Pu et al., 2023; Sahebkar et al., 2023; Wang X. et al., 2025; Wang C. et al., 2025). At present, the use of antiplatelet agents combined with cholesterol-lowering medications, including aspirin and atorvastatin, remains the conventional therapy for atherosclerosis (Castellano et al., 2022; Davidson et al., 2022). Typical treatments in Western medicine for atherosclerosis have shown significant effects in combating atherosclerosis and reducing lipid levels. However, adverse effects, including gastrointestinal discomfort, increased bleeding risk, and potential liver and kidney damage, were observed during treatment, which could restrict their broader clinical use (Thompson, 2016; Ji et al., 2020).

*Amomum tsao-ko Crevost et Lemarie* (T-K) is processed by sun-drying, stir-frying until it turns to charred golden-yellow, and dehulling. The kernel obtained through this process is then termed as Caoguo Ren in Chinese. This herb has a strong, pungent aroma and is considered warm in nature, primarily acting on the spleen and stomach meridians. Moreover, this herb exerts therapeutic effects, including drying dampness, warming the middle jiao, eliminating phlegm, and intercepting malaria. Clinically, it is used to treat epigastric distension and pain, nausea, vomiting, phlegm-associated cough, and malaria, while also alleviating alcohol toxicity and managing halitosis. Studies have reported that its extract possesses pharmacological activities, including the regulation of gastrointestinal function and possessing antibacterial, anti-inflammatory, hypoglycemic, and lipid-regulating effects. In our study, we aimed to evaluate the efficacy of oral administration of T-K in alleviating atherosclerosis in ApoE knockout (ApoE^−/−^) mice. Given the strong connection between gut microbiota and liver function, we further confirmed how T-K exerts its therapeutic effects by reducing hepatic lipid deposition and inflammatory responses. Using 16S rRNA sequencing and histological techniques, we deeply explored the therapeutic mechanisms of T-K for atherosclerosis in the ApoE^−/−^ mice model.

## 2 Materials and methods

### 2.1 Preparation of T-K extract

T-K (batch number: 23060101-1) were procured from Henan Zhangzhongjing Pharmacy Co., Ltd. (Henan, China). Crushed and dried T-K seeds and deionized water were added at a material-to-solvent ratio of 1:20 (g/mL). The extraction was performed by oscillating the mixture in a water bath set to a constant 60 °C for 2 h. After coarse filtration, the filtrate was subjected to centrifugation at 4 °C and 8,000 rpm for 15 min to obtain a clear extract. Subsequently, the extract was concentrated using rotary evaporation and freeze-dried into a powder. The resulting powder was then stored in a sealed container at −20 °C, protecting it from direct light.

### 2.2 Animal model and drug treatments

Male ApoE^−/−^ mice on a C57BL/6 background (6 weeks old; body weight 20 ± 2 g) were purchased from GemPharmatech Co., Ltd. (Soochow, China; SCXK2023-0009). All mice were housed in a specific pathogen-free environment with ad libitum access to food and water, under controlled conditions of 22–24 °C, 45–60% relative humidity, and a 12-h light/dark cycle. All animal experiments were conducted in accordance with the regulations and guidelines of the Animal Welfare and Ethics Committee of Henan University of Chinese Medicine and were approved by the committee (No. DWLLGZR202303167). A total of 38 mice were randomly assigned to individually ventilated cages (four mice/cage). After a week-long adaptation period, the mice were grouped, and the modeling process commenced. Mice in control (Con) group (*n* = 11) were fed low-fat low-sugar (LFLS) control diet (TP 26352; 14% protein, 10% fat, and 72% carbohydrates). Mice in model (Mod) group (*n* = 27) were fed with a high-fat high-cholesterol (HFHC) diet (TP 26304-050; 14% protein, 42% fat, 44% carbohydrates, and 0.5% cholesterol).

After modeling for 12 weeks (Zhang et al., 2021; Wang X. et al., 2025; Wang C. et al., 2025), three mice each from the Mod group and Con group were randomly sacrificed, and the proportion of lipid plaque area to total aortic surface area was quantified using Oil Red O staining (Supplementary Figure 1). Subsequently, 24 mice in from the Mod group were randomly assigned to three subgroups, including Mod group, atorvastatin calcium (ATV) group, and T-K group. The diet of the mice remained unchanged. Con group and Mod group were treated with distilled water, ATV group received 10 mg/kg/d, and the T-K group received 100 mg/kg/d (Zhang et al., 2022). Once-daily intragastric delivery of treatments was carried out over the course of 8 weeks, with a daily dose of 0.1 mL/10 g (Ping et al., 2024). During the final 2 days of experiment, fecal samples were collected, placed into sterile Eppendorf tubes, and subsequently stored at −80 °C. Following the final treatment, serum samples were collected. After pre-cooling normal saline perfusion, the heart and aorta were collected and then fixed in 4% paraformaldehyde. Liver tissues were immediately excised and processed after sacrifice. Part of liver was fixed in 4% paraformaldehyde, while the remaining tissue was rapidly collected and frozen at −80 °C for future use.

### 2.3 Materials and reagents

Lipitor (atorvastatin calcium) was purchased from Pfizer Pharmaceutical Co., Ltd. (New York, United States). Kits for measuring triglyceride (TG, A110-1-1), total cholesterol (TC, A111-1-1), high-density lipoprotein (HDL, A112-1-1), low-density lipoprotein (LDL, A113-1-1), aspartate aminotransferase (AST, C010-2-1), alanine aminotransferase (ALT, C009-2-1), reduced glutathione (GSH, A006-2-1) malondialdehyde (MDA, A003-1-2), and superoxide dismutase (SOD, A001-3-2) were supplied by Nanjing Jiancheng Bioengineering Institute (Nanjing, China). BCA protein assay kit (BCA, PC0020), RIPA lysis buffer, and saline (0.9%) were sourced from Solarbio Biotechnology Co., Ltd. (Beijing, China). Polyformaldehyde (4%) was acquired from Biosharp (Anhui, China). Interleukin-1β (IL-1β, kt21178), interleukin-6 (IL-6, kt99854), tumor necrosis factor-α (TNF-α, kt99985), interleukin-17 (IL-17, kt22800) kits were obtained from Moshake Biotechnology Co., Ltd. (Wuhan, China).

### 2.4 Experimental methods

#### 2.4.1 Assessment of serum and liver biochemical markers

Following collection via enucleation, blood samples were allowed to coagulate at ambient temperature for 40 min. Consequently, samples were centrifuged (3,000 rpm, 10 min, 4 °C) to obtain serum for subsequent analysis. As outlined in prior studies, liver samples were processed into homogenates (Liu F. et al., 2021; Liu L. et al., 2021). The supernatant was then collected for subsequent analysis. Serum levels of TC, TG, HDL, and LDL, as well as liver concentrations of AST, ALT, TC, and TG were determined depending on instructions of the manufacturer.

#### 2.4.2 Assessment of oxidative stress and inflammatory markers in liver and aorta tissue

Aortic tissue samples were homogenized following previously published protocols to obtain tissue homogenates for subsequent analysis (Liu N. et al., 2022; Liu H. et al., 2022). The protein concentration was determined using the BCA assay, with absorbance measured at 562 nm. SOD, GSH activities, and MDA levels in liver and aorta tissue were measured according to the kit instructions. IL-1β, IL-6, TNF-α, and IL-17 were measured in accordance with the manufacturer’s instructions.

#### 2.4.3 Atherosclerotic lesion analysis

After the mice were sacrificed, Oil Red O staining was performed on the full-length aortas of four randomly selected mice per group to assess overall lipid deposition. The aorta was carefully dissected under a stereomicroscope to excise surrounding connective tissue, then opened longitudinally. The entire aorta was stained with Oil Red O solution for 10 min and rinsed with 60% isopropyl alcohol for 2 min to remove excess stain. For aortic root analysis, all eight mice in each group were included. The hearts were collected, fixed in 4% paraformaldehyde, embedded in OCT compound, and cryosectioned at a thickness of 5 μm. The sections were subjected to Oil Red O, hematoxylin and eosin (H&E), and Masson’s trichrome staining. The stained aortic root sections and whole aorta tissues were scanned and photographed separately. Image-Pro Plus 6.0 software was used to measure the lesion area and collagen-positive area in the aortic root, followed by quantitative analysis (Zhou et al., 2012).

#### 2.4.4 Intestinal injury and liver lesion histology (H&E staining)

Liver and intestinal tissues were subjected to stain following the protocol outlined in prior research (Shi et al., 2021; Yang et al., 2022). The severity of hepatic steatosis was assessed using the NASH Activity Score (NAS) system (Liu F. et al., 2021; Liu L. et al., 2021). In addition, histological features, including steatosis, inflammation, and fibrosis, were evaluated.

#### 2.4.5 Analysis based on 16S rRNA gene sequencing

Following the previously established procedure, the target amplification products were acquired and quantified for use in subsequent sequencing (Lu et al., 2020; Dai et al., 2022). Aata analysis and 16S rRNA gene sequencing were carried out following established methods (Wang Q. et al., 2022; Wang Y. et al., 2022). To explore the functional alterations of gut microbiota, predictive functional profiling was performed using PICRUSt2.

#### 2.4.6 Correlation analysis

To explore potential associations, a correlation analysis was conducted linking physiological indicators, oxidative stress levels, inflammatory cytokines, and gut microbial profiles. The expression correlation coefficients were determined using the Pearson correlation coefficient method. Based on these correlations, a network was constructed to visualize the relationships between these factors.

### 2.5 Statistical analysis

Quantitative data are presented as mean ± SD. Data normality was assessed using the Shapiro–Wilk test, and homogeneity of variance was evaluated using Levene’s test. For data that met both assumptions, one-way ANOVA followed by Tukey’s *post hoc* test was used. When the assumption of homogeneity of variance was violated, the Kruskal–Wallis test followed by Dunn’s multiple comparisons test was applied. Statistical analyses were performed using SPSS (version 22.0), and graphs were generated with GraphPad Prism (version 9.5.0). A *p*-value <0.05 was considered statistically significant.

## 3 Results

### 3.1 T-K treatment reduced plaque formation in aorta

**Figure 1:**
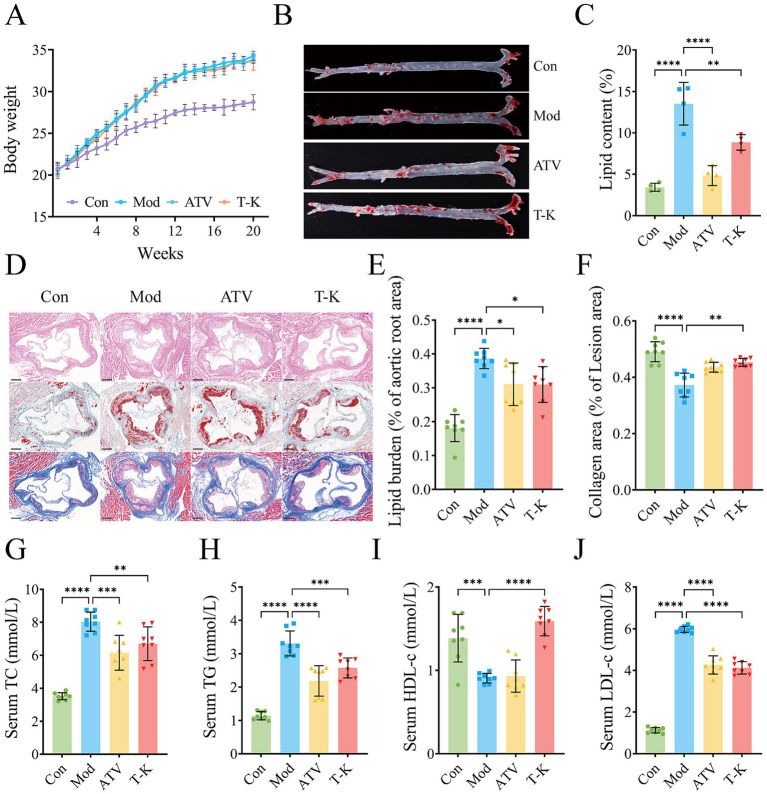
Effects of T-K on development of atherosclerotic lesion. **(A)** Body weight changes of mice in each group after different treatments. **(B)** Representative images of Oil Red O staining of aorta. **(C)** Quantitative analysis of plaque area in entire aorta, *n* = 4. **(D)** Represents H&E, Oil Red O, and Masson staining of aortic root sections (scale bar = 200 μm). **(E)** Percentage of lipid burden in aortic root. **(F)** Percentage of collagen area in lesion area. **(G–J)** Represents content determination of total cholesterol, triglyceride, HDL-c, and LDL-c, respectively (*n* = 8). Data are presented as mean ± SD, ^*^p < 0.05, ^**^p < 0.01, ^***^p < 0.001, and ^****^p < 0.0001, vs. Mod group.

To assess the anti-atherosclerotic properties of T-K, an ApoE^−/−^ mice model of atherosclerosis was established through a HFHC diet (van Vlijmen et al., 1994). The intake of food and water did not differ significantly across all groups (Supplementary Figure 2). After 12 weeks of modeling, mice in HFHC diet group exhibited a more pronounced rise in body weight contrast to the LFLS group. After 8 weeks of administering T-K, body weight continued to increase, but mice in T-K group exhibited a more gradual gain in body weight relative to the Mod group (Figure 1A). To evaluate lipid accumulation in the entire aorta, en face Oil Red O staining was performed. As shown in Figures 1B,C, the Mod group exhibited increased lipid deposition compared to the Con group. Both ATV and T-K treatment significantly reduced lipid content relative to the Mod group, with no significant difference between ATV and T-K groups (*n* = 4). Histological analysis of aortic root sections (*n* = 8) was further conducted (Figure 1D). H&E staining revealed more extensive plaque formation in the Mod group, while the lesion area was reduced in both the ATV and T-K groups. Consistently, Oil Red O staining (Figure 1E) showed a significant increase in lipid burden in the Mod group compared to the Con group, which was attenuated by ATV and T-K treatment. To assess plaque stability, Masson’s trichrome staining was used to evaluate collagen content (Figure 1F). The Mod group exhibited significantly reduced collagen deposition compared to the Con group, whereas both ATV and T-K treatment restored collagen content within the lesions, suggesting improved plaque stability. The results showed that T-K treatment has a significant improvement effect on the progression of atherosclerotic plaque formation in ApoE^−/−^ mice.

### 3.2 T-K regulated lipid metabolism in atherosclerosis mice

Given that atherosclerosis is defined by sustained inflammatory activity in the arterial wall, primarily triggered by lipid accumulation, lipid-lowering therapy remains a fundamental approach in managing atherosclerosis (Libby et al., 2019). Evidence indicates that LDL-C is widely regarded as a causative agent in the onset and progression of CVDs (Penson et al., 2018). As illustrated in Figures 1G–J, serum TC, TG, and LDL levels were elevated, while HDL levels were significantly decreased in the Mod group compared to the Con group (*n* = 8). In contrast, the T-K group exhibited significantly reduced serum TC, TG, and LDL levels along with significantly increased HDL levels when compared to the Mod group. These findings suggest that T-K improved serum lipid profiles and alleviated atherosclerosis in mice.

### 3.3 T-K reduces oxidative stress and inflammation

**Figure 2:**
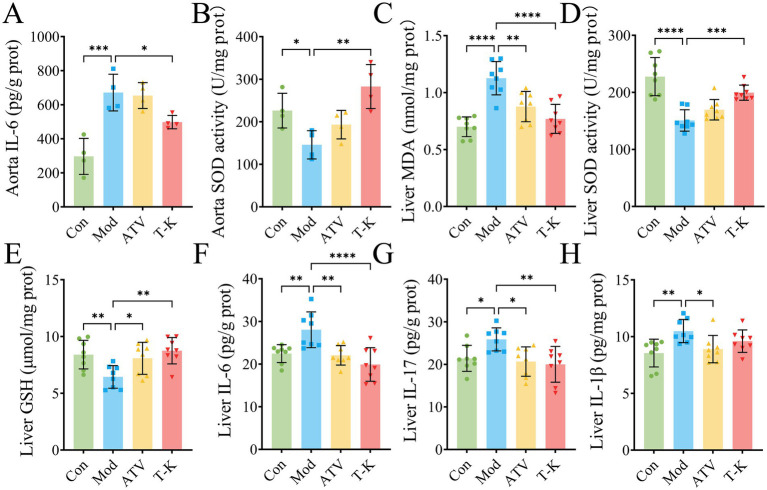
T-K decreases the atherosclerosis by diminishing oxidative stress and inflammation in mice. **(A–D)** Represents the levels of IL-6/SOD activity in aorta and MDA/SOD activity in liver, respectively. **(E–H)** Represents the levels of GSH, IL-6, IL-17, and IL-1β in liver, respectively. Data are presented as mean ± SD, *n* = 8, ^*^p < 0.05, ^**^p < 0.01, ^***^p < 0.001, and ^****^p < 0.0001, vs. Mod group.

The underlying mechanisms of CVDs are closely associated with both oxidative stress and inflammation (Barton et al., 2007; Cheng et al., 2023). Thus, we measured the amounts of MDA, GSH, SOD, TNF-α, IL-6, IL-1β, and IL-17 in liver and abdominal aorta. The results indicated that when compared with LFLS diet of the Con group, HFHC diet substantially raised the levels of MDA (*n* = 4, Supplementary Figure 3A), IL-1β (*n* = 4, Supplementary Figure 3C), TNF-α (*n* = 4, Supplementary Figure 3D), IL-6 (*n* = 4, Figure 2A), and IL-17 (*n* = 4, Supplementary Figure 3E) in aorta of mice at the Mod group. A similar trend was detected in the liver of mice (*n* = 8, Figures 2C–G; Supplementary Figures 3F,G). However, the levels of SOD (*n* = 4, Figure 2B) and GSH (*n* = 4, Supplementary Figure 3B) in aorta of mice in the Mod group were substantially reduced as compared to the Con group. Relative to the Mod group, the T-K group showed certain significance in expression of SOD (*n* = 4, Figure 2B) and IL-6 (*n* = 4, Figure 2A) in aorta. However, it did not show the ability to reduce MDA, SOD, TNF-α, IL-6, IL-1β, and IL-17, and nor did it elevate GSH levels (Figure 2; Supplementary Figure 3B). Test results in mouse livers showed that compared with the Mod group, the T-K group markedly upregulated the expression of MDA (*n* = 8, Figure 2C), IL-6 (*n* = 8, Figure 2F), and IL-17 (*n* = 8, Figure 2G), and substantially elevated SOD levels (*n* = 8, Figure 2D) and GSH (*n* = 8, Figure 2E). No notable alterations were observed in the expression of TNF-α (*n* = 8, Supplementary Figure 3G) and IL-1β (*n* = 8, Supplementary Figure 3F). These findings on oxidative stress and inflammation offer valuable insights for further investigation into the anti-atherosclerotic mechanisms of T-K.

### 3.4 Effects of T-K on histopathology in atherosclerosis mice

**Figure 3:**
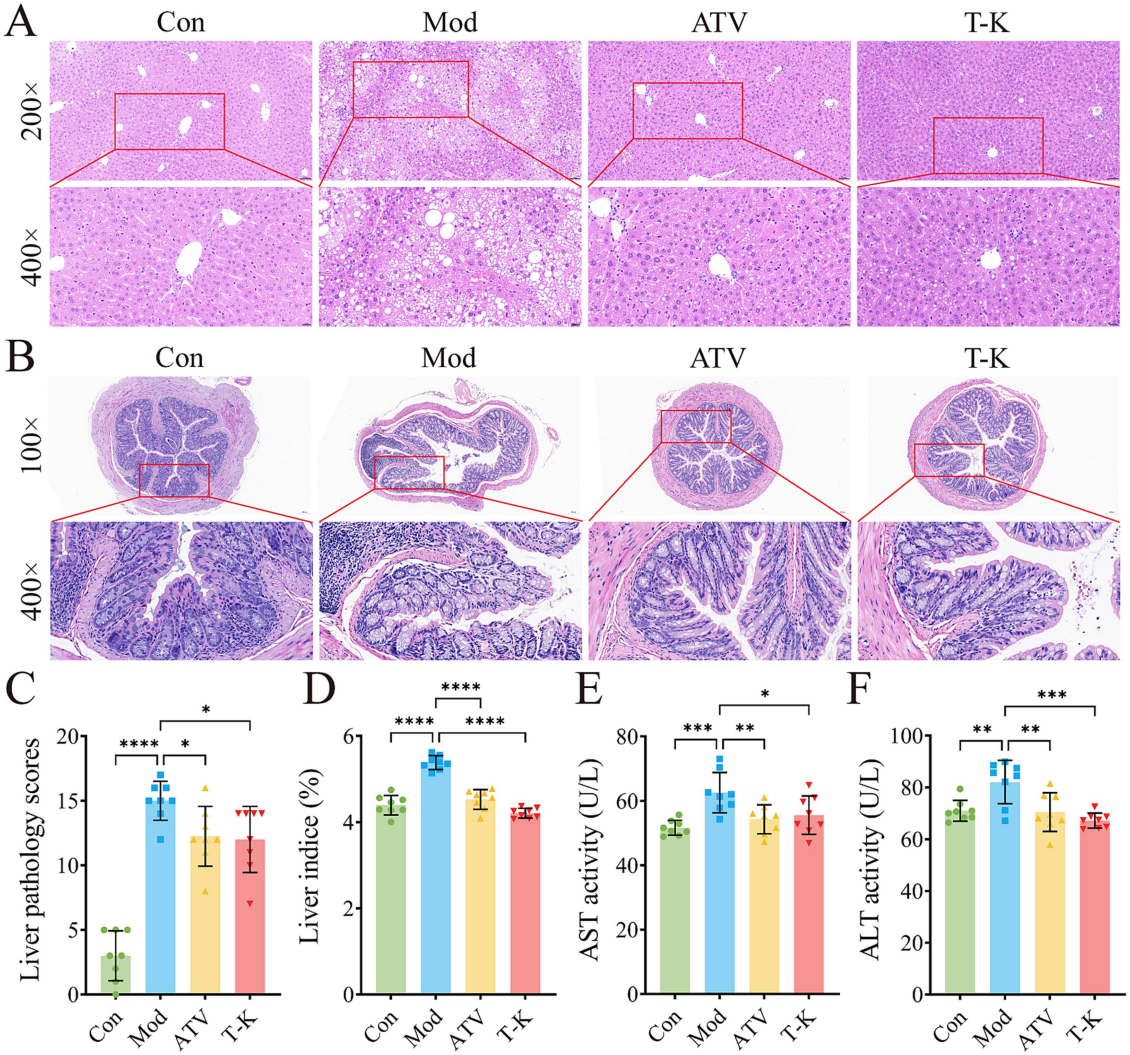
Effects of T-K on histopathology of liver and colon in atherosclerotic mice. **(A)** Represents images of liver H&E staining (scale bar is 50 μm and 20 μm separately). **(B)** Represents images of colon H&E staining (scale bars is 100 μm and 20 μm separately). **(C–F)** Represents liver pathology scores, liver weight indexes, ALT, and AST level, respectively. Data are presented as mean ± SD, *n* = 8, ^*^p < 0.05, ^**^p < 0.01, ^***^p < 0.001, and ^****^p < 0.0001, vs. Mod group.

As shown by H&E stained liver paraffin sections, HFHC-fed atherosclerosis mice displayed notable histopathological changes in the liver (Figure 3A). Liver tissue of the Mod group mice exhibited extensive hepatocellular steatosis, with cytoplasmic vacuoles of varying sizes observed within the hepatocytes. Ballooning degeneration was evident, characterized by swollen hepatocytes with balloon-like morphology, nucleus centralization, and cytoplasmic vacuolization. Compression of liver sinusoids was noted, and liver cord architecture was disrupted. In addition, focal infiltration of granulocytes and lymphocytes was occasionally observed within liver lobules, suggesting the presence of inflammatory responses and tissue damage. In contrast, the ATV and T-K groups had minimal fat vacuoles, and histological scoring of liver steatosis indicated significant improvement in the pathological features of atherosclerosis in mice (Figures 3A,C). Relative to the Con group, a significant increase in the liver weight index was observed in the Mod group of atherosclerotic mice that was effectively suppressed by T-K and atorvastatin treatments (Figure 3D). Given that T-K’s safety requires thorough evaluation, we assessed hepatotoxicity markers following 2 months of drug administration. Specifically, we measured the levels of ALT and AST (*n* = 8, Figures 3E,F).

H&E staining of mouse colon showed that intestinal gland structure of the Con group was complete, regularly arranged, and the cells were clearly layered, with no evident pathological changes (Figure 3B). In comparison, colon gland morphology of the Mod group changed significantly, with expanded glandular spaces, disordered cell arrangement, edema in some areas, and mild inflammatory cell infiltration, suggesting obvious damage or inflammation in intestine. The ATV group showed that colon gland morphology was restored, glands were arranged more regularly, and inflammatory cell infiltration was reduced. Relative to the Mod group, the intestinal tissue structure of the T-K group was relatively complete, glands were tightly arranged, and no significant inflammatory cell infiltration was found, indicating that the drug may have a strong intestinal protective effect and can effectively inhibit the inflammatory response. Overall, the ATV group and the T-K group showed a more significant effect in reducing intestinal inflammatory response and might have contributed positively by modulating the abundance and composition of intestinal microorganisms.

### 3.5 T-K altered the diversity of the gut microbiota

**Figure 4:**
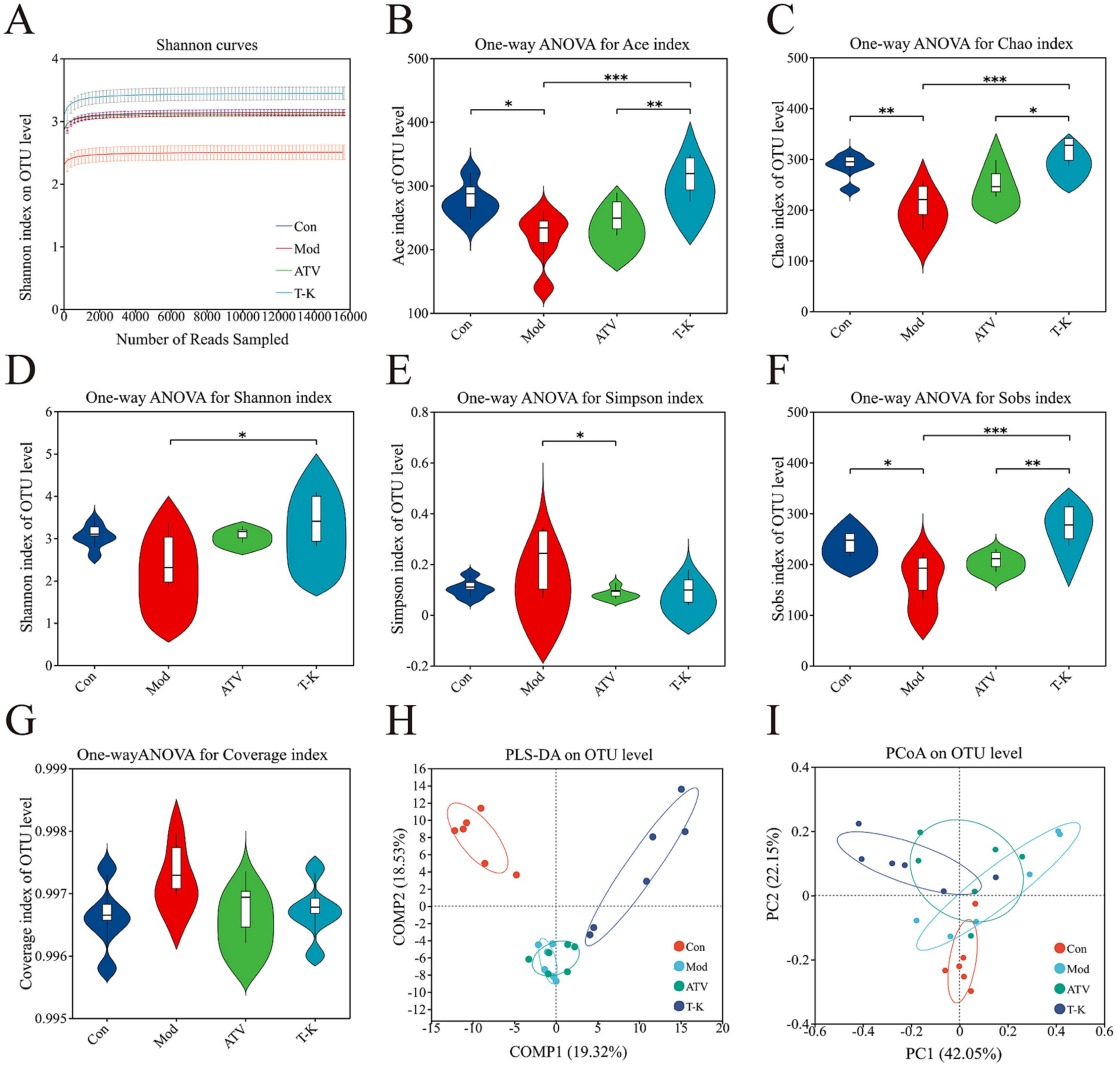
Effects of T-K on microbial diversity and community structure in atherosclerotic mice. **(A)** Shannon curves. **(B–G)** Represents the Ace index (reflecting microbial richness), Chao index (indicating species abundance), Shannon index (showing overall microbial diversity), Simpson index (measuring microbial evenness), Sobs index (representing species richness), and Coverage index (indicating sequencing depth and completeness), respectively. **(H,I)** Represents the PLS-DA plot of OTU level (demonstrating distinct clustering of groups) and the PCoA plot of OTU level (showing differences in microbial community composition across the group), respectively (*n* = 6, ^*^p < 0.05, ^**^p < 0.01, and ^***^p < 0.001, vs. Mod group).

Alterations in microbial community structure and function have been implicated in numerous disease processes. There is substantial evidence linking gut microbiota-derived processes to the development of atherosclerosis (Witkowski et al., 2020). It is now understood that the gut microbiota influences the progression of atherosclerosis through various mechanisms, for instance, lipid metabolism or other related processes. To investigate these changes, fecal microbiome compositions were analyzed to identify differences and assess the extent of microbiome alterations across different experimental groups. Following the method described earlier, fecal samples were processed for 16S rRNA sequencing (*n* = 6). In Figure 4A, the Shannon curves show a tendency to level off, signifying that the test captures most of the microbial composition in the sample.

#### 3.5.1 Alpha diversity

Multiple different alpha diversity analyses, including species observed (Sobs), Shannon–Wiener index (Shannon), Simpson’s diversity index (Simpson), Chao1 estimator (Chao1), abundance-based coverage estimator (ACE), and coverage indexes, could reflect the community diversity of gut microbiota. Microbial richness was evaluated using the Sobs, Chao1, and Ace indexes, while microbial diversity was assessed with the Shannon and Simpson indexes. As shown in Figures 4B–E, T-K not only substantially elevated the Ace, Chao1, Sobs, and Shannon indexes of atherosclerosis mice, but the index of T-K group generally exhibited higher values compared to the ATV group, indicating that T-K performed better in terms of microbial richness and diversity and restored the gut microbiota’s alpha diversity in mice. The Simpson index of the Mod group is significantly higher, indicating that certain species dominate within this group (Figure 4F). The coverage index can assess the sequencing depth, and the values of these groups approximated 1, indicating successful sequencing and nearly complete coverage of all species within the samples (Figure 4G).

#### 3.5.2 Beta diversity

We further examined the beta diversity to assess how gut microbiota composition differed among the groups. Partial least squares discriminant analysis (PLS-DA) and principal coordinates analysis (PCoA) results (Figures 4H,I) demonstrated a clear distinction among the groups regarding their gut microbiota composition. Notably, as observed in the figure, the groups receiving the T-K treatment formed a distinct cluster, with a clear separation from both the Mod and ATV groups. This indicates that the gut microbiota changes, following the T-K treatment, may exhibit a unique pattern, distinct from the alterations observed in the statin-treated groups.

#### 3.5.3 Gut microbiota abundance

**Figure 5:**
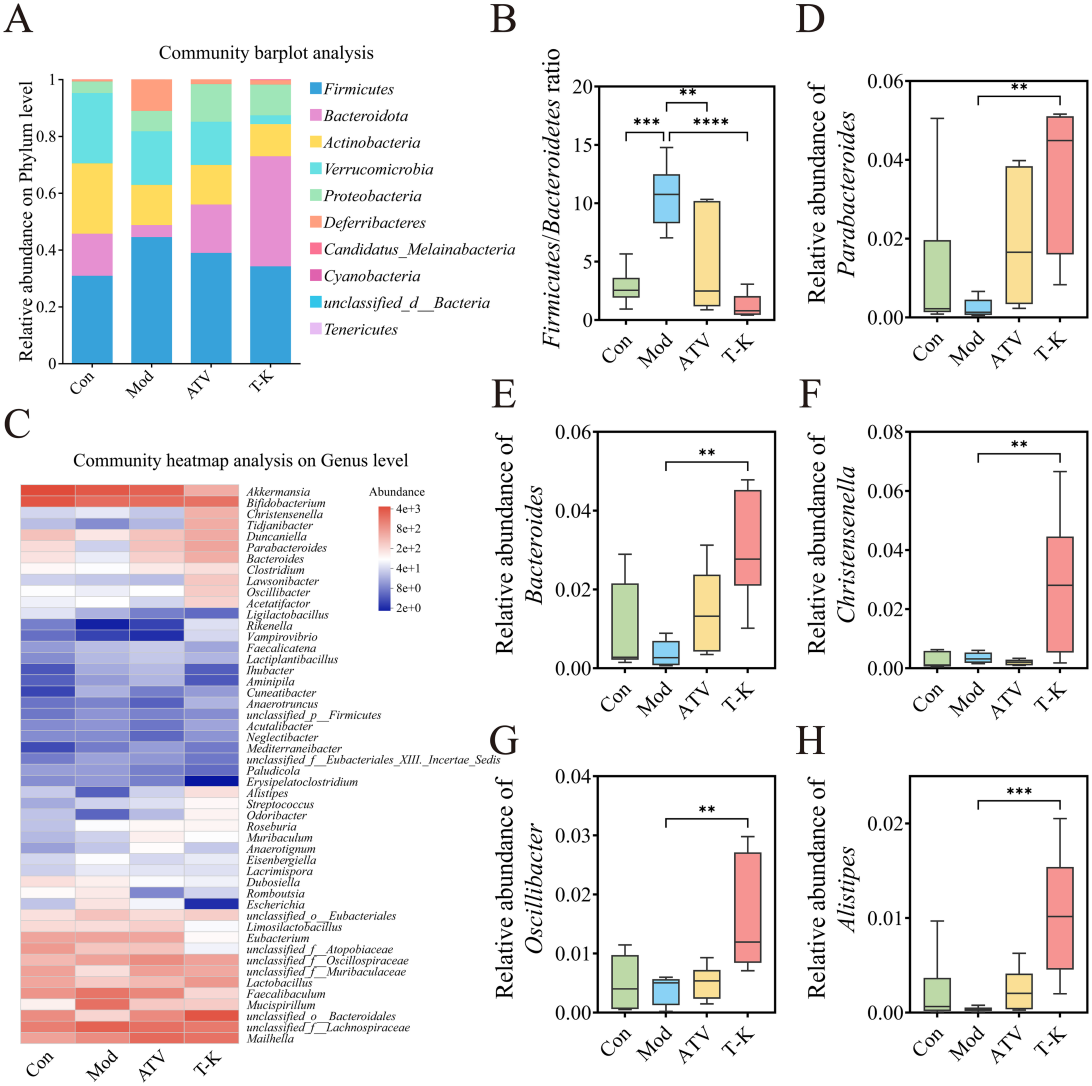
Effects of T-K on gut microbiota composition in atherosclerotic mice. **(A)** Community bar plot analysis at the phylum level. **(B)** *Firmicutes/Bacteroidetes* ratio. **(C)** Top 50 genera heatmap. **(D–H)** Represents the relative abundance of *Parabacteroides*, *Bacteroides*, *Christensenella*, *Oscillibacter*, and *Alistipes*, respectively. Data are presented as mean ± SD, *n* = 6, ^**^p < 0.01, ^***^p < 0.001, and ^****^p < 0.0001, vs. Mod group.

**Figure 6:**
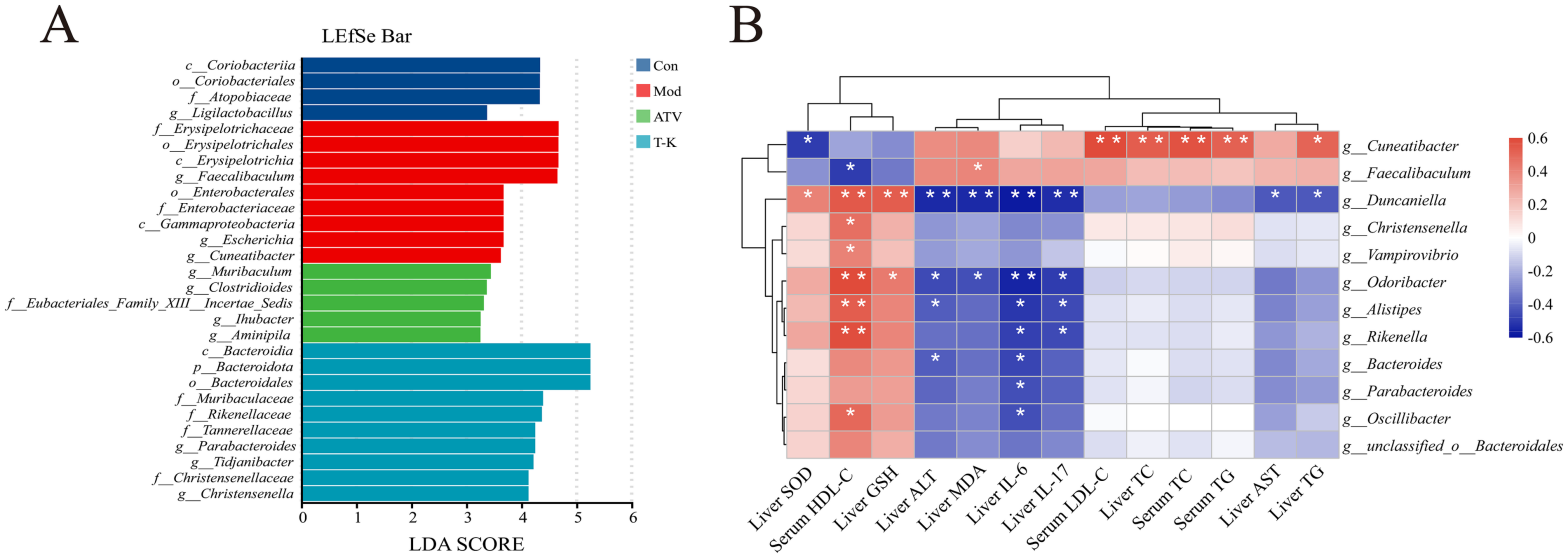
Effects of T-K on gut microbiota and its correlation with liver and serum parameters in atherosclerotic mice. **(A)** LEfSe bar plot displaying the LDA scores for the most discriminative taxa between the groups. **(B)** Heatmap showing the correlation between microbial genera and various liver and serum parameters (^*^p < 0.05 and ^**^p < 0.01).

We assessed the relative abundance of taxa at different levels within each group, revealing specific changes in the gut microbiota across the different mouse groups. Figure 5A demonstrated the changes among groups at the phylum levels. *Firmicutes* and *Bacteroidota* were predominant phyla, and T-K decreased both the relative abundance of *Firmicutes* and the ratio of *Firmicutes* to *Bacteroidetes* (Figure 5B), in contrast, the relative abundance of *Bacteroidota* and *Candidatus Melainabacteria* was significantly elevated (*n* = 6, Supplementary Figures 4A,B). Our results demonstrated that T-K corrected intestinal dysbiosis through analysis of F/B ratio for gut microbial dysbiosis (Santisteban et al., 2017). The heatmap of the top 50 genera at the genus level clearly depicts the alterations in gut microbiota composition within each group (Figure 5C). The analysis identified 12 genera that exhibited significant alterations in their abundance (Supplementary Figure 5; Figure 6). We utilized the Disbiome database (Janssens et al., 2018) to extract data on the relationship of these genera with the development of atherosclerotic diseases in humans (Supplementary Table 1). Within available evidence, we identified five genera that showed significant changes in abundance before and after the T-K treatment and were closely associated with atherosclerotic diseases, namely, *Parabacteroides*, *Bacteroides*, *Christensenella*, *Oscillibacter*, and *Alistipes*. Overall, their abundance markedly elevated after the T-K treatment (Figures 5D–H), while the abundance of *Faecalibaculum*, which belongs to the *Lachnospiraceae* family, was significantly decreased.

#### 3.5.4 Gut microbial correlation analysis

T-K treatment significantly altered the gut microbiota composition and abundance in atherosclerotic mice. Concurrently, aortic atherosclerotic area, lipid levels, liver oxidative stress, and body inflammation were improved, showing a favorable trend of change. To figure out whether these favorable changes were associated with gut microbiota alterations, we conducted a correlation analysis between microbiota composition and the above indicators to reveal their potential links. Differences in the fecal microbiota were examined using linear discriminant analysis effect size (LEfSe) (Figure 6A). Pearson’s correlation analysis was then performed between the selected genera showing significant differences and the measured indicators (Figure 6B). Among them, relative abundances of *Cuneatibacter* and *Faecalibaculum* were significantly enriched in the Mod group. In addition, *Cuneatibacter* exhibited a significant correlation with serum LDL-C, liver TC, serum TC, and serum TG concentration, and showed a positive association with liver TG levels, while showing an inverse association with liver SOD. *Faecalibaculum* was inversely associated with serum HDL-C levels and exhibited a marked positive relationship with liver MDA concentration. The proportion of *Duncaniella* also displayed significant positive relationships with SOD, liver GSH, and serum HDL-C concentrations. Furthermore, *Duncaniella’s* abundance displayed a significant inverse relationship with hepatic ALT, liver MDA, liver IL-17, and liver IL-6 concentrations, and was also negatively correlated with liver AST and liver TG concentrations. Supplementation of T-K significantly enriched the relative abundance of *Christensenella* and *Parabacteroides*. *Christensenella* and *Vampirovibrio* showed positive associations with serum HDL-C concentrations. *Parabacteroides* as well as *Oscillibacter* were negatively associated with liver IL-6 concentrations, while *Oscillibacter* was positively linked positively to serum HDL-C concentrations. *Odoribacter* demonstrated a positive association with liver GSH concentration and serum HDL-C concentration, but exhibited an inverse association with liver ALT, liver MDA, liver IL-17 concentration, and significantly negatively correlated with liver IL-6 concentration. *Alistipes* displayed a positive relationship with serum HDL-C concentrations while showing an inverse relationship with liver ALT, liver IL-17, and liver IL-6 concentrations. *Bacteroides* showed inverse correlations with liver ALT and IL-6 concentrations.

These correlations highlight the interactions between different microbiota and various biomarkers associated with liver function, blood lipids, and inflammation. Collectively, the present findings suggest that the gut microbiota has close associations with these parameters, and alterations in microbial structure and abundance could exert a critical influence on the progression and treatment of atherosclerosis.

#### 3.5.5 Gut microbial functional enrichment analysis

As for functional prediction analysis, PICRUSt2 was used to perform the KEGG pathway annotation and enrichment analysis. At Level 2, a total of 43 functional categories were identified, encompassing major categories such as metabolism, genetic information processing, environmental information processing, cellular processes, organismal systems, and human diseases. Overall, after multiple testing correction, most categories showed no significant differences (FDR >0.05). However, as illustrated in Supplementary Figure 6A, nominal significance and intergroup mean trends revealed that the Mod group exhibited general reductions in metabolism-related subclasses, including carbohydrate, lipid, coenzyme and vitamin, and amino acid metabolism, whereas infection- and immune-related categories were relatively elevated. Following intervention, both ATV and T-K showed restorative effects on metabolic and signaling categories and exhibited a regressive trend in cell death-related processes. At the Level 3, 287 pathways were identified, among which 49 showed nominal differences (*p* < 0.05). Supplementary Figure 6B demonstrates that in the Mod group, pathways related to lipid and energy metabolism, including fatty acid degradation, peroxisome, linoleic acid metabolism, one-carbon pool by folate, thiamine metabolism, PPAR signaling, and insulin signaling, were decreased, whereas these pathways were restored to varying extents in the T-K and ATV groups. Conversely, pathways associated with cell death and stress responses, such as apoptosis, ferroptosis, and p53 signaling, were enriched in the Mod group but regressed after T-K intervention. Collectively, these findings indicated that T-K is at least comparable to ATV in reconstructing metabolic homeostasis and suppressing stress responses, and in certain pathways, its effects are even more pronounced.

## 4 Discussion

Extensive research has demonstrated that lipid accumulation, oxidative stress, and inflammation responses are key pathogenic mechanisms underlying atherosclerosis (Liu N. et al., 2022; Liu H. et al., 2022). This study systematically evaluated the effects of T-K in ApoE^−/−^ mice with atherosclerosis. T-K significantly reduced lipid deposition in the aortic sinus and increased collagen content within plaques, suggesting improved plaque stability. It also alleviated inflammation in both the liver and aorta. By lowering serum TC, TG, and LDL-C levels and increasing HDL-C, T-K exerted hepatoprotective effects and improved colonic morphology. In addition, T-K enhanced gut microbiota diversity and reshaped microbial community structure. LEfSe analysis identified specific bacterial taxa enriched in the T-K group, potentially associated with its regulatory effects on the gut microbiota. Compared to HFHC diet-fed mice, the T-K treatment led to distinct shifts in gut microbial composition, suggesting that T-K may optimize gut microecology through targeted microbiota modulation, thereby offering new insights into atherosclerosis management. These microbial changes may also contribute to the regulation of host metabolism, immune function, and inflammatory responses. Moreover, correlation heatmap analysis revealed significant associations between specific bacterial taxa and hepatic or serum biomarkers, including SOD, GSH, ALT, MDA, HDL-C, LDL-C, and TG. Collectively, these findings support the therapeutic potential of T-K in the multifactorial management of atherosclerosis, in line with previous reports (Yoshida et al., 2018; Lavillegrand et al., 2024).

Through analysis of gut microbiota abundance and composition, the significantly different species in T-K group include the phyla *Bacteroidota*, *Candidatus Melainabacteria*, and genera *Parabacteroides*, *Bacteroides*, *Christensenella*, *Oscillibacter*, and *Alistipes*. Previous studies have shown that the genus *Bacteroides* is less abundant in individuals with coronary artery disease (CAD) compared to those with risk factors but without CAD or healthy controls. In particular, reduced abundance of *Bacteroides vulgatus* and *Bacteroides dorei* is associated with elevated fecal LPS levels in CAD patients, whereas supplementation with these strains alleviated inflammation and atherosclerotic lesion formation in mice. These findings are consistent with the gut microbiota alterations observed in our experimental mice (Yoshida et al., 2018). It is associated with parameters such as liver IL-6 level, which suggests that gut microbiota might play an indirect role in the pathological process of atherosclerosis by promoting inflammatory responses. After drug treatment in hyperlipidemic mice, levels of *Candidatus Melainabacteria* in microbiota markedly elevated, further confirming the crucial importance of intestinal microbiota to host health (Lu et al., 2022). Emerging evidence suggesting that a *Ganoderma meroterpene* derivative alleviates obesity-related atherosclerosis via enrichment of intestinal *Parabacteroides*. At the same time, supplementation with viable *Parabacteroides* in ApoE^−/−^ mice fed a high-fat diet can reduce the atherosclerotic plaques (Qiao et al., 2022). Evidence suggests that CAD is associated with reductions in *Bacteroidetes* and *Alistipes* populations. In particular, the abundance of *Alistipes* is negatively correlated with liver IL-17 and liver IL-6 levels, while positively correlated with serum HDL-C, indicating that dysbiosis of gut microbiota promotes endothelial inflammation and progression of atherosclerosis (Choroszy et al., 2022). In our study, treatment with T-K extract led to an increased abundance of *Alistipes* in atherosclerotic mice. Similarly, Wang et al. reported that *Ginkgo biloba* extract supplementation in Western diet-fed Ldlr^−/−^ mice elevated *Alistipes*, a shift associated with improved intestinal barrier function and alleviated atherosclerotic lesions (Wang Q. et al., 2022; Wang Y. et al., 2022). These consistent observations suggest that modulation of *Alistipes* may represent a common microbial response to botanical interventions in atherosclerosis, although its functional implications warrant further clarification (Liang et al., 2024). In our study, the relative abundance of *Faecalibaculum* was markedly elevated in atherosclerotic mice but significantly reduced following the treatment with T-K extract. This finding is in line with recent reports indicating that the abundance of *Faecalibaculum* is associated with bile acid dysregulation and activation of the intestinal FXR-FGF19 axis, thereby contributing to lipid metabolism disorders and atherosclerosis progression (Xu et al., 2023). Conversely, suppression of *Faecalibaculum* by T-K extract may attenuate bile acid-driven dysmetabolism, reduce pro-inflammatory signaling, and ultimately alleviate atherosclerotic lesions (Liang et al., 2024). Nevertheless, the associations of *Christensenella* and *Oscillibacter* with atherosclerosis remain incompletely characterized in current research.

Analysis of the functional prediction results using PICRUSt2 indicated that T-K may ameliorate atherosclerosis by enhancing lipid metabolism pathways, restoring the balance of PPAR and insulin signaling, and suppressing the excessive activation of apoptosis and ferroptosis, thereby exerting coordinated effects on multiple pathological processes. However, it should be noted that the KEGG functional annotation and pathway analysis in this study were primarily based on predictive results, which entails certain uncertainties, particularly as the enrichment significance at Levels 2 and 3 was limited after multiple-testing correction. Therefore, future studies should incorporate transcriptomic, proteomic, or metabolomic data for multi-omics integration to strengthen the reliability of the conclusions.

Recent studies have highlighted the significant role of the gut-liver axis in modulating systemic and hepatic inflammation. Gut microbiota dysbiosis increases intestinal permeability, allowing bacterial products such as lipopolysaccharides (LPS) to translocate into the portal circulation, which subsequently activates hepatic immune responses and promotes liver inflammation and oxidative stress (Anand and Mande, 2022). Moreover, short-chain fatty acids (SCFAs), produced by beneficial gut microbes, exert anti-inflammatory effects and contribute to intestinal barrier integrity (Verhaar et al., 2020). These findings support our observation that T-K improves liver inflammation in parallel with microbiota remodeling. Although our current study is based on correlation analysis, the consistency with previous mechanistic studies suggests a plausible link between microbial modulation and hepatic improvement. In addition, microbiota-derived metabolites such as TMAO and bile acids have been implicated in both cholesterol metabolism and vascular inflammation, indicating that the observed improvement in atherosclerosis may also be partially microbiota-mediated (Mao et al., 2024; Buchynskyi et al., 2025). Notably, the study by Zhu et al. (2020) directly demonstrates that *Alisma orientale* beverage reduces atherosclerotic plaque formation by modulating gut microbiota and lowering TMAO levels in ApoE^−/−^ mice. Together, these findings underscore the importance of gut-derived metabolites such as TMAO in linking intestinal dysbiosis to vascular pathology and suggest that targeting TMAO may be a promising strategy in future anti-atherosclerotic therapies (Liu et al., 2023).

Although this study provides new insights into the atheroprotective effects of T-K via modulation of the gut–liver axis, several limitations should be taken into consideration. The absence of a control + T-K group (healthy mice receiving T-K without disease induction) limits the evaluation of baseline effects of T-K on gut microbiota and host metabolism under physiological conditions. Although no overt toxicity was observed during the 8-week intervention, the long-term safety profile of T-K remains unclear; comprehensive toxicological assessments, including chronic toxicity and organ-specific evaluations, will be important in future studies. Histological analysis was restricted to Oil Red O staining, without cellular characterization of lesions such as smooth muscle cells, macrophages, and leukocytes. These parameters are essential for better understanding the impact of T-K on plaque composition. The relatively small number of animals included in histological evaluation may also reduce statistical power. Moreover, while PLS-DA and LEfSe analyses revealed significant alterations in gut microbiota composition, the absence of microbiota-depleted (pseudo-germ-free) controls prevents definitive conclusions about causality. These limitations highlight the need for more comprehensive experimental designs in future research to fully elucidate the therapeutic potential and underlying mechanisms of T-K.

Till date, the pharmacological research progress of T-K has been systematically summarized by Lu et al. (2022). At least 209 compounds have been isolated and identified from T-K, yet most of these components remain functionally uncharacterized and require further investigation. In the present study, the phytochemical constituents of T-K were identified and are listed in Supplementary Table 2. We have also added a brief summary of several key bioactive constituents that may contribute to the observed pharmacological effects of T-K. For instance, hyperoside (quercetin-3-O-galactoside) has been reported to inhibit LPS-induced NF-κB activation in macrophages, thereby reducing the production of TNF-α, IL-6, and nitric oxide (Kim et al., 2011). It also improves lipid profiles and alleviates hepatic steatosis in high-fat diet-induced models via activation of the PPARγ and FXR/LXRα pathways (Wang et al., 2021). Vanillin and its oxidative metabolite vanillic acid exhibit strong antioxidant and anti-inflammatory activities by scavenging reactive oxygen species and suppressing proinflammatory cytokines, in part through NF-κB inhibition (Kafali et al., 2024). Chlorogenic acid is known to modulate endothelial function, inhibit platelet aggregation, and regulate lipid metabolism, providing cardiovascular protection (Fuentes et al., 2014). In addition, quercetin, a well-studied flavonoid, has been shown to reduce atherosclerotic plaque area in ApoE^−/−^ mice by attenuating oxidative stress and upregulating HO-1 and eNOS expression (Shen et al., 2013). Although some of these components have been functionally validated in *in vitro* or animal studies, clinical evidence remains scarce. This represents an important future direction, as clinical validation will be essential for translating these findings into therapeutic applications (Zhang et al., 2022). Furthermore, the precise mechanisms through which T-K alleviates atherosclerosis remain incompletely understood. Therefore, future studies should focus on the isolation and characterization of individual bioactive compounds, *in vivo* functional validation, and ultimately clinical trials. Such investigations are expected to provide deeper insight into the therapeutic potential of T-K.

In summary, this study indicates that T-K may exert anti-atherosclerotic effects by regulating blood lipid levels, alleviating inflammation, and reshaping the composition of gut microbiota. However, our current research findings are primarily correlational rather than causal, and it is very important to further clarify the mechanisms. In future work, approaches such as fecal microbiota transplantation, antibiotic-induced microbiota depletion, and multi-omics integration will be essential to determine whether the gut microbiota directly mediates the therapeutic effects of T-K. In particular, metabolomics focusing on gut microbiota-derived metabolites, including SCFAs, bile acids and TMAO, will provide mechanistic insight into the microbiota-host interaction. In addition, isolation and functional characterization of individual bioactive compounds will help differentiate the contribution of phytochemical constituents and microbiota-derived mechanisms. By addressing these questions, the therapeutic potential of T-K can be fully elucidated, providing a stronger foundation for its possible use in the prevention and treatment of atherosclerosis and related metabolic disorders.

## Data Availability

The sequencing data have been deposited in the NCBI Sequence Read Archive (SRA) under accession number PRJNA1334592 (BioProject). The dataset includes 24 BioSamples and their corresponding SRA accessions, which can be accessed at https://www.ncbi.nlm.nih.gov/bioproject/PRJNA1334592.

## Glossary

T-K: *Amomum tsao-ko Crevost et Lemarie*
H&E: Hematoxylin-eosin
TG: Triglycerides
TC: Total cholesterol
LDL-C: Low-density lipoprotein cholesterol
HDL-C: High-density lipoprotein cholesterol
AST: Aspartate aminotransferase
ALT: Alanine aminotransferase
IL-1β: Interleukin-1β
IL-6: Interleukin-6
IL-17: Interleukin-17
TNF-α: Tumor necrosis factor-α
SOD: Superoxide dismutase
MDA: Malondialdehyde
GSH: Glutathione
HFHC: High-fat high-cholesterol diet
LFLS: Low-fat low-sugar diet
ATV: Atorvastatin
ELISA: Enzyme-linked immunosorbent assay
LEfSe: Linear discriminant analysis effect size
Sobs: Species observed
Shannon: Shannon–Wiener index
Simpson: Simpson’s diversity index
Chao1: Chao1 estimator
ACE: Abundance-based coverage estimator
PLS-DA: Partial least squares discriminant analysis
PCoA: Principal coordinates analysis

## References

[ref1] Anand S. Mande S. S. (2022). Host-microbiome interactions: gut-liver axis and its connection with other organs. npj Biofilms Microbiomes 8:89. doi: 10.1038/s41522-022-00352-6, PMID: 36319663 PMC9626460

[ref2] Barton M. Minotti R. Haas E. (2007). Inflammation and atherosclerosis. Circ. Res. 101, 750–751. doi: 10.1161/CIRCRESAHA.107.162487, PMID: 17932331

[ref3] Björkegren J. L. M. Lusis A. J. (2022). Atherosclerosis: recent developments. Cell 185, 1630–1645. doi: 10.1016/j.cell.2022.04.004, PMID: 35504280 PMC9119695

[ref4] Buchynskyi M. Kamyshna I. Halabitska I. Petakh P. Kunduzova O. Oksenych V.. (2025). Unlocking the gut-liver axis: microbial contributions to the pathogenesis of metabolic-associated fatty liver disease. Front. Microbiol. 16:1577724. doi: 10.3389/fmicb.2025.1577724, PMID: 40351307 PMC12061941

[ref5] Castellano J. M. Pocock S. J. Bhatt D. L. Quesada A. J. Owen R. Fernandez-Ortiz A.. (2022). Polypill strategy in secondary cardiovascular prevention. N. Engl. J. Med. 387, 967–977. doi: 10.1056/NEJMoa2208275, PMID: 36018037

[ref6] Cheng C. Zhang J. Li X. Xue F. Cao L. Meng L.. (2023). NPRC deletion mitigated atherosclerosis by inhibiting oxidative stress, inflammation and apoptosis in ApoE knockout mice. Signal Transduct. Target. Ther. 8:290. doi: 10.1038/s41392-023-01560-y, PMID: 37553374 PMC10409771

[ref7] Choroszy M. Sobieszczańska B. Litwinowicz K. Łaczmański Ł. Chmielarz M. Walczuk U.. (2022). Co-toxicity of endotoxin and indoxyl sulfate, gut-derived bacterial metabolites, to vascular endothelial cells in coronary arterial disease accompanied by gut dysbiosis. Nutrients 14:424. doi: 10.3390/nu14030424, PMID: 35276782 PMC8840142

[ref8] Dai X. Gu Y. Guo J. Huang L. Cheng G. Peng D.. (2022). Clinical breakpoint of apramycin to swine salmonella and its effect on ileum flora. Int. J. Mol. Sci. 23:1424. doi: 10.3390/ijms23031424, PMID: 35163350 PMC8835974

[ref9] Davidson K. W. Barry M. J. Mangione C. M. Cabana M. Chelmow D. Coker T. R.. (2022). Aspirin use to prevent cardiovascular disease: US preventive services task force recommendation statement. JAMA 327, 1577–1584. doi: 10.1001/jama.2022.4983, PMID: 35471505

[ref10] Falk E. (2006). Pathogenesis of atherosclerosis. J. Am. Coll. Cardiol. 47, C7–C12. doi: 10.1016/j.jacc.2005.09.068, PMID: 16631513

[ref11] Fuentes E. Caballero J. Alarcón M. Rojas A. Palomo I. (2014). Chlorogenic acid inhibits human platelet activation and thrombus formation. PLoS One 9:e90699. doi: 10.1371/journal.pone.0090699, PMID: 24598787 PMC3944540

[ref12] Herrington W. Lacey B. Sherliker P. Armitage J. Lewington S. (2016). Epidemiology of atherosclerosis and the potential to reduce the global burden of atherothrombotic disease. Circ. Res. 118, 535–546. doi: 10.1161/CIRCRESAHA.115.307611, PMID: 26892956

[ref13] Janssens Y. Nielandt J. Bronselaer A. Debunne N. Verbeke F. Wynendaele E.. (2018). Disbiome database: linking the microbiome to disease. BMC Microbiol. 18:50. doi: 10.1186/s12866-018-1197-5, PMID: 29866037 PMC5987391

[ref14] Ji W. Jiang T. Sun Z. Teng F. Ma C. Huang S.. (2020). The enhanced pharmacological effects of modified traditional Chinese medicine in attenuation of atherosclerosis is driven by modulation of gut microbiota. Front. Pharmacol. 11:546589. doi: 10.3389/fphar.2020.546589, PMID: 33178012 PMC7593568

[ref15] Kafali M. Finos M. A. Tsoupras A. (2024). Vanillin and its derivatives: a critical review of their anti-inflammatory, anti-infective, wound-healing, neuroprotective, and anti-cancer health-promoting benefits. Nutraceuticals 4, 522–561. doi: 10.3390/nutraceuticals4040030

[ref16] Kim S.-J. Um J.-Y. Lee J.-Y. (2011). Anti-inflammatory activity of hyperoside through the suppression of nuclear factor-κB activation in mouse peritoneal macrophages. Am. J. Chin. Med. 39, 171–181. doi: 10.1142/S0192415X11008737, PMID: 21213407

[ref17] Lavillegrand J.-R. Al-Rifai R. Thietart S. Guyon T. Vandestienne M. Cohen R.. (2024). Alternating high-fat diet enhances atherosclerosis by neutrophil reprogramming. Nature 634, 447–456. doi: 10.1038/s41586-024-07693-6, PMID: 39232165 PMC12019644

[ref18] Liang X. Zheng X. Wang P. Zhang H. Ma Y. Liang H.. (2024). *Bifidobacterium animalis* subsp. *lactis* F1-7 alleviates lipid accumulation in atherosclerotic mice via modulating bile acid metabolites to downregulate intestinal FXR. J. Agric. Food Chem. 72, 2585–2597. doi: 10.1021/acs.jafc.3c05709, PMID: 38285537

[ref19] Libby P. Buring J. E. Badimon L. Hansson G. K. Deanfield J. Bittencourt M. S.. (2019). Atherosclerosis. Nat. Rev. Dis. Primers 5:56. doi: 10.1038/s41572-019-0106-z 31420554

[ref20] Liu N. Qin H. Cai Y. Li X. Wang L. Xu Q.. (2022). Dynamic trafficking patterns of IL-17-producing γδ T cells are linked to the recurrence of skin inflammation in psoriasis-like dermatitis. EBioMedicine 82:104136. doi: 10.1016/j.ebiom.2022.104136, PMID: 35785620 PMC9256835

[ref21] Liu F. Shan S. Li H. Shi J. Hao R. Yang R.. (2021). Millet shell polyphenols prevent atherosclerosis by protecting the gut barrier and remodeling the gut microbiota in ApoE^−/−^ mice. Food Funct. 12, 7298–7309. doi: 10.1039/d1fo00991e, PMID: 34169953

[ref22] Liu L. Yuan Y. Tao J. (2021). Flavonoid-rich extract of *Paeonia lactiflora* petals alleviate d-galactose-induced oxidative stress and restore gut microbiota in ICR mice. Antioxidants 10:1889. doi: 10.3390/antiox10121889, PMID: 34942992 PMC8698645

[ref23] Liu J. Zhou S. Wang Y. Liu J. Sun S. Sun Y.. (2023). ZeXieYin formula alleviates TMAO-induced cognitive impairment by restoring synaptic plasticity damage. J. Ethnopharmacol. 314:116604. doi: 10.1016/j.jep.2023.116604, PMID: 37178985

[ref24] Liu H. Zhu L. Chen L. Li L. (2022). Therapeutic potential of traditional Chinese medicine in atherosclerosis: a review. Phytother. Res. 36, 4080–4100. doi: 10.1002/ptr.7590, PMID: 36029188

[ref25] Lu X. Han B. Deng X. Deng S.-Y. Zhang Y.-Y. Shen P.-X.. (2020). Pomegranate peel extract ameliorates the severity of experimental autoimmune encephalomyelitis via modulation of gut microbiota. Gut Microbes 12:1857515. doi: 10.1080/19490976.2020.1857515, PMID: 33382357 PMC7751635

[ref26] Lu X. Qin Z. Bai L.-X. Ge W.-B. Li J.-Y. Yang Y.-J. (2022). Untargeted lipidomics and metagenomics reveal the mechanism of aspirin eugenol ester relieving hyperlipidemia in ApoE^−/−^ mice. Front. Nutr. 9:1030528. doi: 10.3389/fnut.2022.1030528, PMID: 36618709 PMC9815714

[ref27] Mao Y. Kong C. Zang T. You L. Wang L.-S. Shen L.. (2024). Impact of the gut microbiome on atherosclerosis. mLife 3, 167–175. doi: 10.1002/mlf2.12110, PMID: 38948150 PMC11211673

[ref28] Penson P. E. Long D. L. Howard G. Toth P. P. Muntner P. Howard V. J.. (2018). Associations between very low concentrations of low density lipoprotein cholesterol, high sensitivity C-reactive protein, and health outcomes in the reasons for geographical and racial differences in stroke (REGARDS) study. Eur. Heart J. 39, 3641–3653. doi: 10.1093/eurheartj/ehy533, PMID: 30165636 PMC6195947

[ref29] Ping L. Zhi-Ming L. Bi-Shan Z. Lei Z. Bo Y. Yi-Chun Z.. (2024). S-propargyl-cysteine promotes the stability of atherosclerotic plaque via maintaining vascular muscle contractile phenotype. Front. Cell Dev. Biol. 11:1291170. doi: 10.3389/fcell.2023.1291170, PMID: 38328305 PMC10847265

[ref30] Pu Y. Cheng C. K. Zhang H. Luo J.-Y. Wang L. Tomlinson B.. (2023). Molecular mechanisms and therapeutic perspectives of peroxisome proliferator-activated receptor α agonists in cardiovascular health and disease. Med. Res. Rev. 43, 2086–2114. doi: 10.1002/med.21970, PMID: 37119045

[ref31] Qiao S. Liu C. Sun L. Wang T. Dai H. Wang K.. (2022). Gut *Parabacteroides merdae* protects against cardiovascular damage by enhancing branched-chain amino acid catabolism. Nat. Metab. 4, 1271–1286. doi: 10.1038/s42255-022-00649-y, PMID: 36253620

[ref32] Roth G. A. Mensah G. A. Johnson C. O. Addolorato G. Ammirati E. Baddour L. M.. (2020). Global burden of cardiovascular diseases and risk factors, 1990–2019. J. Am. Coll. Cardiol. 76, 2982–3021. doi: 10.1016/j.jacc.2020.11.010, PMID: 33309175 PMC7755038

[ref33] Sahebkar A. Foroutan Z. Katsiki N. Jamialahmadi T. Mantzoros C. S. (2023). Ferroptosis, a new pathogenetic mechanism in cardiometabolic diseases and cancer: is there a role for statin therapy? Metabolism 146:155659. doi: 10.1016/j.metabol.2023.155659, PMID: 37442270

[ref34] Santisteban M. M. Qi Y. Zubcevic J. Kim S. Yang T. Shenoy V.. (2017). Hypertension-linked pathophysiological alterations in the gut. Circ. Res. 120, 312–323. doi: 10.1161/CIRCRESAHA.116.309006, PMID: 27799253 PMC5250568

[ref35] Shen Y. Ward N. C. Hodgson J. M. Puddey I. B. Wang Y. Zhang D.. (2013). Dietary quercetin attenuates oxidant-induced endothelial dysfunction and atherosclerosis in apolipoprotein E knockout mice fed a high-fat diet: a critical role for heme oxygenase-1. Free Radic. Biol. Med. 65, 908–915. doi: 10.1016/j.freeradbiomed.2013.08.185, PMID: 24017971

[ref36] Shi S. Verstegen M. M. A. Roest H. P. Ardisasmita A. I. Cao W. Roos F. J. M.. (2021). Recapitulating cholangiopathy-associated necroptotic cell death in vitro using human cholangiocyte organoids. Cell. Mol. Gastroenterol. Hepatol. 13, 541–564. doi: 10.1016/j.jcmgh.2021.10.009 34700031 PMC8688721

[ref37] Thompson P. D. (2016). What to believe and do about statin-associated adverse effects. JAMA 316, 1969–1970. doi: 10.1001/jama.2016.16557, PMID: 27838726

[ref38] Vaduganathan M. Mensah G. A. Turco J. V. Fuster V. Roth G. A. (2022). The global burden of cardiovascular diseases and risk: a compass for future health. J. Am. Coll. Cardiol. 80, 2361–2371. doi: 10.1016/j.jacc.2022.11.005, PMID: 36368511

[ref39] van Vlijmen B. J. van den Maagdenberg A. M. Gijbels M. J. van der Boom H. HogenEsch H. Frants R. R.. (1994). Diet-induced hyperlipoproteinemia and atherosclerosis in apolipoprotein E3-leiden transgenic mice. J. Clin. Invest. 93, 1403–1410. doi: 10.1172/JCI117117, PMID: 8163645 PMC294153

[ref40] Verhaar B. J. H. Prodan A. Nieuwdorp M. Muller M. (2020). Gut microbiota in hypertension and atherosclerosis: a review. Nutrients 12:2982. doi: 10.3390/nu12102982, PMID: 33003455 PMC7601560

[ref41] Wang X. Cui J. Gu Z. Guo L. Liu R. Guo Y.. (2025). Aged garlic oligosaccharides modulate host metabolism and gut microbiota to alleviate high-fat and high-cholesterol diet-induced atherosclerosis in ApoE^−/−^ mice. Food Chem. 463:141409. doi: 10.1016/j.foodchem.2024.141409, PMID: 39326312

[ref42] Wang Q. He Y. Li X. Zhang T. Liang M. Wang G.. (2022). *Lactobacillus reuteri* CCFM8631 alleviates hypercholesterolaemia caused by the paigen atherogenic diet by regulating the gut microbiota. Nutrients 14:1272. doi: 10.3390/nu14061272, PMID: 35334930 PMC8953203

[ref43] Wang C. Lv J. Yang M. Fu Y. Wang W. Li X.. (2025). Recent advances in surface functionalization of cardiovascular stents. Bioact. Mater. 44, 389–410. doi: 10.1016/j.bioactmat.2024.10.025, PMID: 39539518 PMC11558551

[ref44] Wang S. Sheng F. Zou L. Xiao J. Li P. (2021). Hyperoside attenuates non-alcoholic fatty liver disease in rats via cholesterol metabolism and bile acid metabolism. J. Adv. Res. 34, 109–122. doi: 10.1016/j.jare.2021.06.001, PMID: 35024184 PMC8655136

[ref45] Wang Y. Xu Y. Xu X. Wang H. Wang D. Yan W.. (2022). *Ginkgo biloba* extract ameliorates atherosclerosis via rebalancing gut flora and microbial metabolism. Phytother. Res. 36, 2463–2480. doi: 10.1002/ptr.7439, PMID: 35312112

[ref46] Witkowski M. Weeks T. L. Hazen S. L. (2020). Gut microbiota and cardiovascular disease. Circ. Res. 127, 553–570. doi: 10.1161/CIRCRESAHA.120.316242, PMID: 32762536 PMC7416843

[ref47] Xu H. Fang F. Wu K. Song J. Li Y. Lu X.. (2023). Gut microbiota-bile acid crosstalk regulates murine lipid metabolism via the intestinal FXR-FGF19 axis in diet-induced humanized dyslipidemia. Microbiome 11:262. doi: 10.1186/s40168-023-01709-5, PMID: 38001551 PMC10675972

[ref48] Yang X. Yu H. Fu J. Guo H.-H. Han P. Ma S.-R.. (2022). Hydroxyurea ameliorates atherosclerosis in ApoE^−/−^ mice by potentially modulating Niemann–Pick C1-like 1 protein through the gut microbiota. Theranostics 12, 7775–7787. doi: 10.7150/thno.76805, PMID: 36451858 PMC9706578

[ref49] Yoshida N. Emoto T. Yamashita T. Watanabe H. Hayashi T. Tabata T.. (2018). *Bacteroides vulgatus* and *Bacteroides dorei* reduce gut microbial lipopolysaccharide production and inhibit atherosclerosis. Circulation 138, 2486–2498. doi: 10.1161/CIRCULATIONAHA.118.033714, PMID: 30571343

[ref50] Zhang L. Li Y. Ma X. Liu J. Wang X. Zhang L.. (2021). Ginsenoside Rg1-notoginsenoside R1-protocatechuic aldehyde reduces atherosclerosis and attenuates low-shear stress-induced vascular endothelial cell dysfunction. Front. Pharmacol. 11:588259. doi: 10.3389/fphar.2020.588259, PMID: 33568993 PMC7868340

[ref51] Zhang X.-F. Tang Y.-J. Guan X.-X. Lu X. Li J. Chen X.-L.. (2022). Flavonoid constituents of *Amomum tsao-ko Crevost et Lemarie* and their antioxidant and antidiabetic effects in diabetic rats—*in vitro* and *in vivo* studies. Food Funct. 13, 437–450. doi: 10.1039/d1fo02974f 34918725

[ref52] Zhou Q. Mei Y. Shoji T. Han X. Kaminski K. Taeg G.. (2012). ROCK2 deficiency in bone marrow-derived cells leads to increased cholesterol efflux and decreased atherosclerosis. Circulation 126, 2236–2247. doi: 10.1161/CIRCULATIONAHA.111.086041 23011471 PMC3807088

[ref53] Zhu B. Zhai Y. Ji M. Wei Y. Wu J. Xue W.. (2020). Alisma orientalis beverage treats atherosclerosis by regulating gut microbiota in ApoE^−/−^ mice. Front. Pharmacol. 11:570555. doi: 10.3389/fphar.2020.570555, PMID: 33101028 PMC7545905

