# Supplementary material for: Amomum tsao-ko Crevost et Lemarie extract targets the gut-liver axis to combat atherosclerosis in ApoE−/− mice
Source: Front Microbiol. 2025 Oct 31;16:1641035. doi: 10.3389/fmicb.2025.1641035 (PMC12617299; doi:10.3389/fmicb.2025.1641035)
Supplement: Supplementary file 1 [file Supplementary_file_1.docx]

***Supplementary Material***


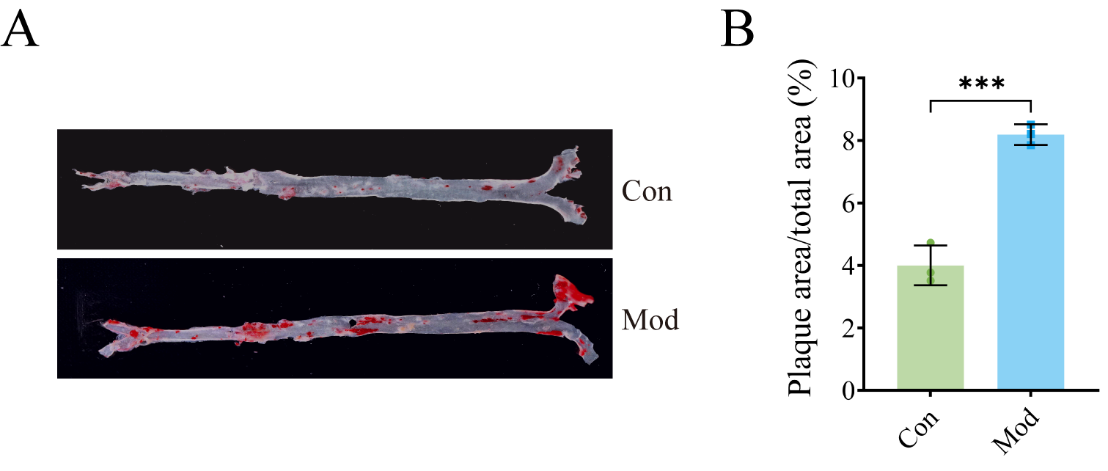


**Supplementary Figure 1. Pathological evidence of the successful establishment of atherosclerotic mice model.**

(A) Representative images of oil red O staining of aorta in Con and Mod mice (red regions indicate lipid plaques). (B) Quantitative analysis of plaque area/total area (%), showing a significant increase in atherosclerotic plaques in Mod group (^***^*P*<0.001).


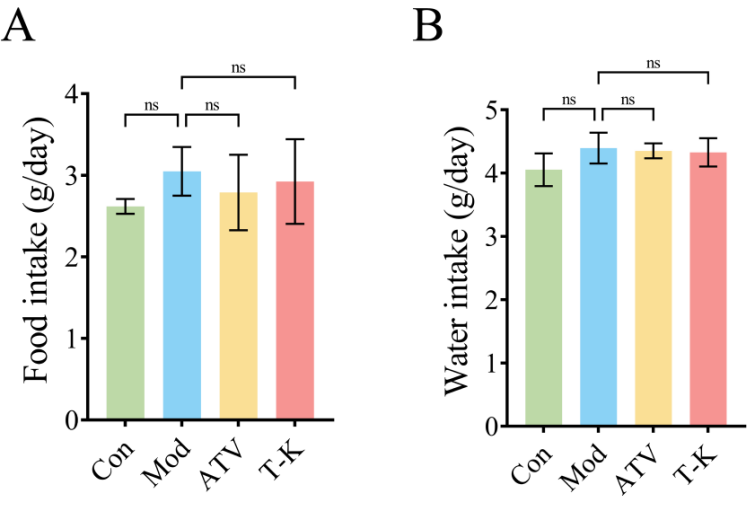


**Supplementary Figure 2. Food and water intake of mice during the experiment.**

(A-B) Represents the T-K effect on food and water intake of mice in each group, separately.


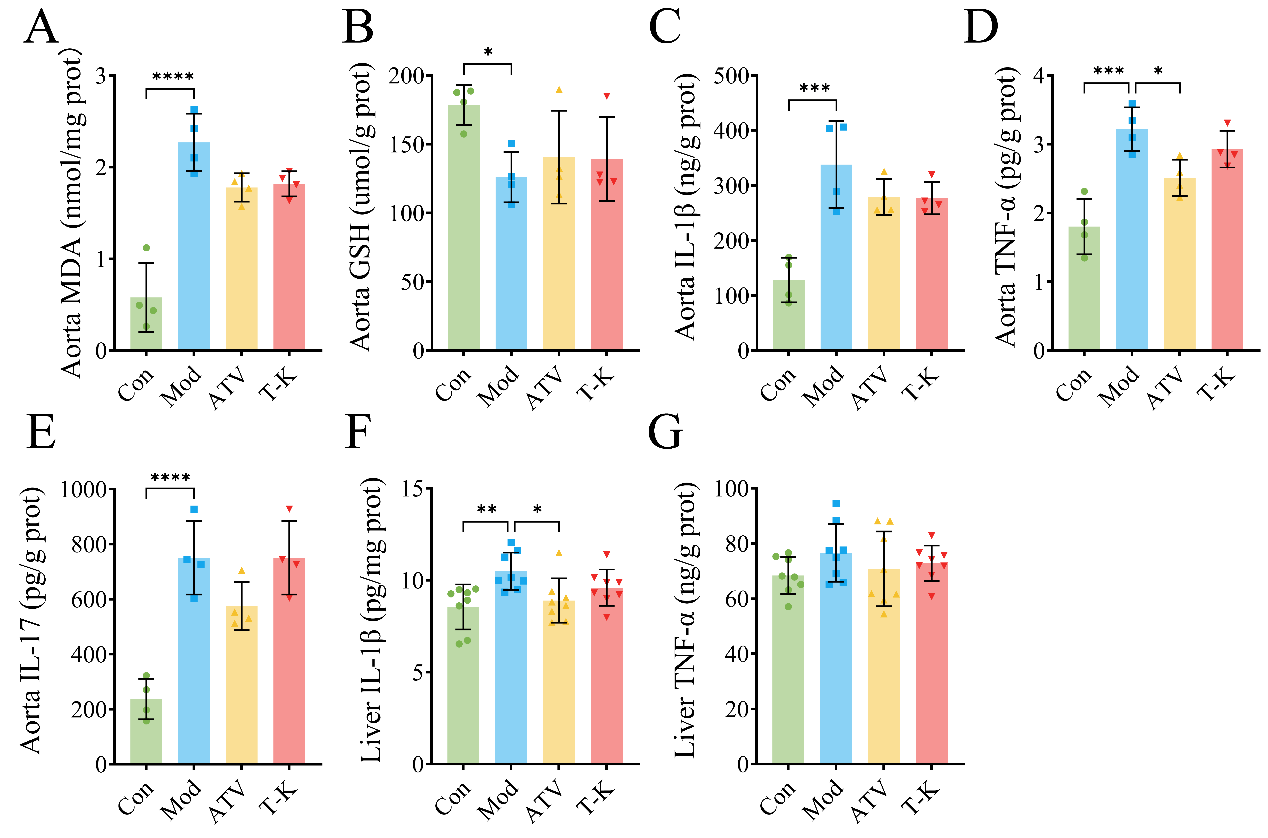


**Supplementary Figure 3. Effects of T-K on inflammatory and oxidative stress markers in atherosclerotic mice.**

(A-E) Represents the levels of MDA, GSH, IL-1β, TNF-α, IL-17 in aorta, respectively; (F-G) Represents the level of IL-1β and TNF-α in liver (vs Mod group).


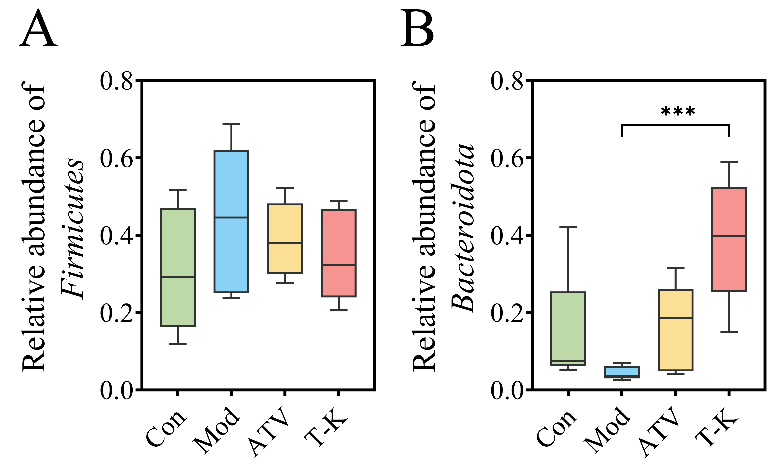


**Supplementary Figure 4. Effects of T-K on relative abundance of *Firmicutes* and *Bacteroidota* in atherosclerotic mice.**

(A-B) Represents the relative abundance of *Firmicutes* and *Bacteroidota* across the groups, separately (vs Mod group, ^***^*P*<0.001).


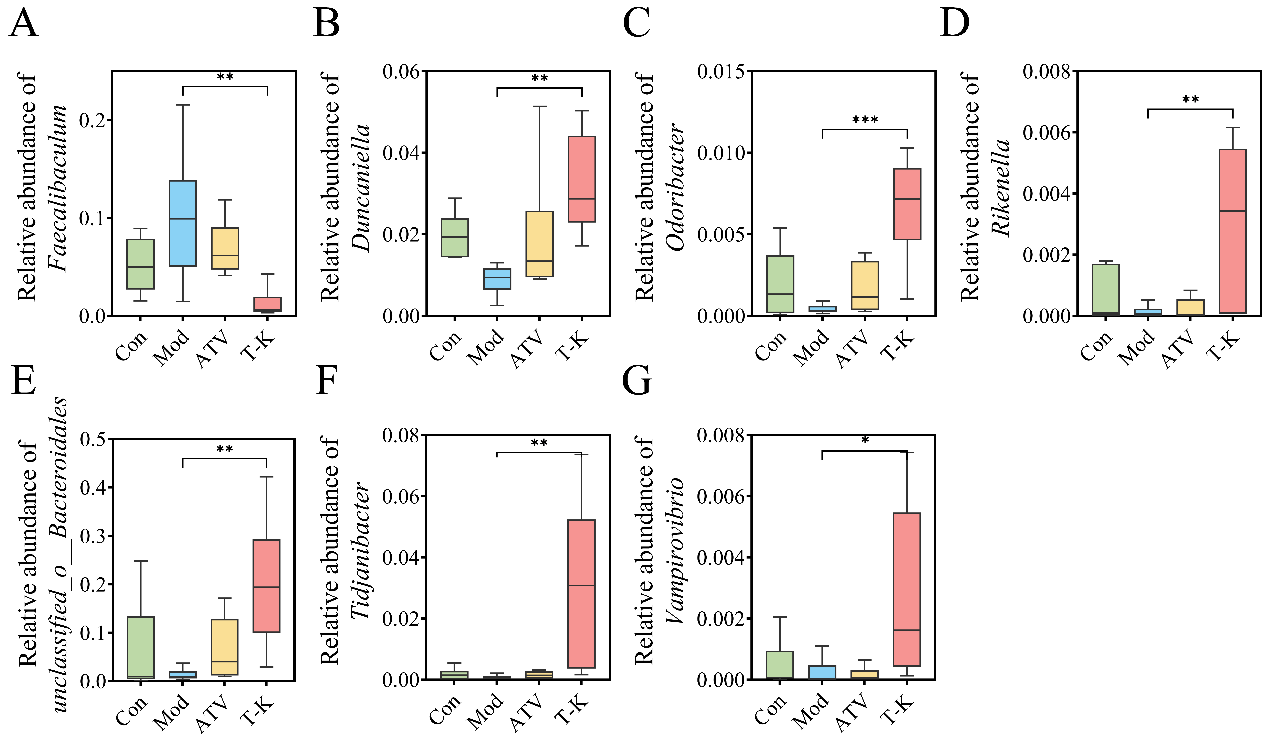


**Supplementary Figure 5. Effects of T-K on relative abundance of specific genera in atherosclerotic mice.**

(A-G) Represents the relative abundance of *Faecalibaculum*, *Duncaniella*, *Odoribacter*, *Rikenella*, *unclassified_o_Bacteroidales*, *Tjidjiabacter*, *Vampirovibrio*, respectively (vs Mod group, ^*^*P*<0.05, ^**^*P*<0.01, ^***^*P*<0.001).


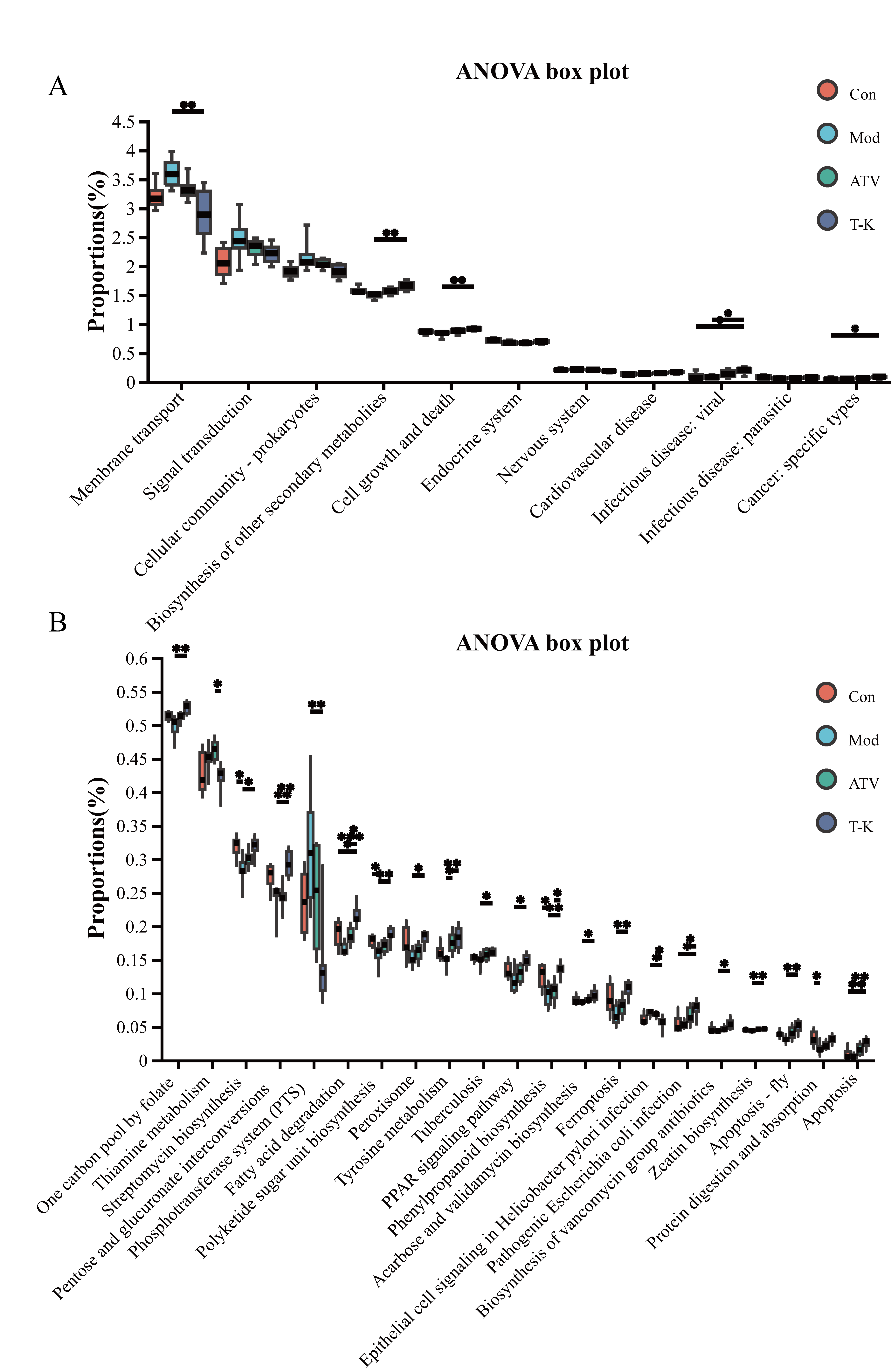
**Supplementary Figure 6. KEGG functional prediction based on PICRUSt2.**

(A) Relative abundance of 43 Level-2 categories, showing decreased metabolic functions and elevated infection/immune-related categories in the model group, with partial restoration by ATV and T-K. (B) Analysis of significantly enriched Level-3 pathways (p < 0.05), highlighting alterations in lipid metabolism, one-carbon metabolism, PPAR/insulin signaling, and cell-death-related pathways, and their correction by drug treatments

**Supplementary Table 1 Different bacteria associated with lipid metabolism and atherosclerosis disease**

| **Organism** | **Diseases** | **Qualitative outcome** | **Method** | **Type** | **Host** | **Reference** |
| --- | --- | --- | --- | --- | --- | --- |
| *Parabacteroides* | Coronary heart disease | Reduced | 16S rRNA sequencing | Faeces | Human | (Zhang et al., 2019) |
|  | Hypertension | Elevated | Shotgun sequencing | Faeces | Human | (Yan et al., 2017) |
|  | Coronary heart disease | Elevated | MiSeq sequencing | Faeces | Human | (Kehrmann et al., 2019) |
|  | Coronary artery disease | Elevated | 16S rRNA sequencing | Faeces | Human | (Liu et al., 2019a) |
| *Bacteroides* | Atherosclerosis | Reduced | 16S rRNA sequencing | Faeces | Human | (Jie et al., 2017) |
|  | Hypertension | Reduced | Shotgun sequencing | Faeces | Human | (Kim et al., 2018) |
|  | Coronary artery disease | Elevated | 16S rRNA sequencing | Faeces | Human | (Liu et al., 2019b) |
|  | Cardiac valve calcification | Reduced | 16S rRNA sequencing | Faeces | Human | (Liu et al., 2019b) |
| *Christensenellaceae* | Coronary artery disease | Reduced | 16S rDNA pyrosequencing | Faeces | Human | (Toya et al., 2020) |
|  | Coronary artery disease | Elevated | 16S rRNA sequencing | Faeces | Human | (Zheng et al., 2020) |
| *Lachnospiraceae* | Non-alcoholic fatty liver disease | Elevated | 16S rRNA sequencing | Faeces | Human | (Adams et al., 2020) |
| *Oscillibacter* | Hypertension | Reduced | Metagenomic sequencing | Faeces | Human | (Li et al., 2017) |
|  | Non-alcoholic fatty liver disease | Reduced | 16S rRNA sequencing | Faeces | Human | (Jiang et al., 2015a) |
| *Alistipes* | Non-alcoholic fatty liver disease | Reduced | 16S rRNA sequencing | Faeces | Human | (Jiang et al., 2015b) |
|  | Atherosclerosis | Reduced | 16S rRNA sequencing | Faeces | Human | (Jie et al., 2017) |
| *Odoribacter* | Non-alcoholic fatty liver disease | Reduced | 16S rRNA sequencing | Faeces | Human | (Jiang et al., 2015a) |
|  | Coronary artery disease | Elevated | 16S rRNA sequencing | Faeces | Human | (Zheng et al., 2020) |
| *Rikenellaceae* | Non-alcoholic fatty liver disease | Reduced | 16S rRNA sequencing | Faeces | Human | (Dong et al., 2020) |

**References for Supplementary Table 1:**

Adams, L. A., Wang, Z., Liddle, C., Melton, P. E., Ariff, A., Chandraratna, H., et al. (2020). Bile acids associate with specific gut microbiota, low-level alcohol consumption and liver fibrosis in patients with non-alcoholic fatty liver disease. *Liver Int.* 40, 1356–1365. doi: 10.1111/liv.14453

Dong, T. S., Katzka, W., Lagishetty, V., Luu, K., Hauer, M., Pisegna, J., et al. (2020). A microbial signature identifies advanced fibrosis in patients with chronic liver disease mainly due to NAFLD. *Sci. Rep.* 10, 2771. doi: 10.1038/s41598-020-59535-w

Jiang, W., Wu, N., Wang, X., Chi, Y., Zhang, Y., Qiu, X., et al. (2015a). Dysbiosis gut microbiota associated with inflammation and impaired mucosal immune function in intestine of humans with non-alcoholic fatty liver disease. *Sci. Rep.* 5, 8096. doi: 10.1038/srep08096

Jiang, W., Wu, N., Wang, X., Chi, Y., Zhang, Y., Qiu, X., et al. (2015b). Dysbiosis gut microbiota associated with inflammation and impaired mucosal immune function in intestine of humans with non-alcoholic fatty liver disease. *Sci. Rep.* 5, 8096. doi: 10.1038/srep08096

Jie, Z., Xia, H., Zhong, S.-L., Feng, Q., Li, S., Liang, S., et al. (2017). The gut microbiome in atherosclerotic cardiovascular disease. *Nat. Commun.* 8, 845. doi: 10.1038/s41467-017-00900-1

Kehrmann, J., Menzel, J., Saeedghalati, M., Obeid, R., Schulze, C., Holzendorf, V., et al. (2019). Gut microbiota in human immunodeficiency virus–infected individuals linked to coronary heart disease. *J. Infect. Dis.* 219, 497–508. doi: 10.1093/infdis/jiy524

Kim, S., Goel, R., Kumar, A., Qi, Y., Lobaton, G., Hosaka, K., et al. (2018). Imbalance of gut microbiome and intestinal epithelial barrier dysfunction in patients with high blood pressure. *Clin. Sci.* 132, 701–718. doi: 10.1042/CS20180087

Li, J., Zhao, F., Wang, Y., Chen, J., Tao, J., Tian, G., et al. (2017). Gut microbiota dysbiosis contributes to the development of hypertension. *Microbiome* 5, 14. doi: 10.1186/s40168-016-0222-x

Liu, Z., Li, J., Liu, H., Tang, Y., Zhan, Q., Lai, W., et al. (2019a). The intestinal microbiota associated with cardiac valve calcification differs from that of coronary artery disease. *Atherosclerosis* 284, 121–128. doi: 10.1016/j.atherosclerosis.2018.11.038

Liu, Z., Li, J., Liu, H., Tang, Y., Zhan, Q., Lai, W., et al. (2019b). The intestinal microbiota associated with cardiac valve calcification differs from that of coronary artery disease. *Atherosclerosis* 284, 121–128. doi: 10.1016/j.atherosclerosis.2018.11.038

Toya, T., Corban, M. T., Marrietta, E., Horwath, I. E., Lerman, L. O., Murray, J. A., et al. (2020). Coronary artery disease is associated with an altered gut microbiome composition. *PLOS ONE* 15, e0227147. doi: 10.1371/journal.pone.0227147

Yan, Q., Gu, Y., Li, X., Yang, W., Jia, L., Chen, C., et al. (2017). Alterations of the gut microbiome in hypertension. *Front. Cell. Infect. Microbiol.* 7. doi: 10.3389/fcimb.2017.00381

Zhang, Y., Xu, J., Wang, X., Ren, X., and Liu, Y. (2019). Changes of intestinal bacterial microbiota in coronary heart disease complicated with nonalcoholic fatty liver disease. *BMC Genomics* 20, 862. doi: 10.1186/s12864-019-6251-7

Zheng, Y.-Y., Wu, T.-T., Liu, Z.-Q., Li, A., Guo, Q.-Q., Ma, Y.-Y., et al. (2020). Gut microbiome-based diagnostic model to predict coronary artery disease. *J. Agric. Food Chem.* 68, 3548–3557. doi: 10.1021/acs.jafc.0c00225

**Supplementary Methods:**

The UHPLC-MS/MS analysis was performed using a Vanquish UHPLC system coupled to a Q Exactive HFX Orbitrap mass spectrometer (Thermo Fisher Scientific, USA). Chromatographic separation was achieved on a Waters HSS T3 column (100 × 2.1 mm, 1.8 μm) with a mobile phase consisting of 0.1% formic acid in water (solvent A) and 0.1% formic acid in acetonitrile (solvent B). The flow rate was 0.3 mL/min and the injection volume was 2 μL. The column temperature was maintained at 40°C. A gradient elution was applied as follows: 0-1 min, 100% A; 1-12 min, linear gradient to 5% A; 12-13 min, held at 5% A; 13.1-17 min, re-equilibrated to 100% A.

Mass spectrometric detection was conducted using a heated electrospray ionization (HESI) source operating in both positive and negative ion modes. Full MS/ddMS2 acquisition was employed with a scan range of m/z 70-1050, MS1 resolution of 70,000, and MS2 resolution of 17,500. The spray voltage was set at +3000 V/ −2800 V, with a capillary temperature of 350°C and ion transfer tube temperature of 320°C. Sheath gas and auxiliary gas were set at 40 and 10 arbitrary units, respectively.

Raw data were processed using Progenesis QI software for peak detection, retention time alignment, normalization, and compound identification. Metabolites were annotated by matching accurate mass, MS/MS fragmentation patterns, and retention times against a proprietary traditional Chinese medicine (TCM) MS/MS spectral library (Shanghai Sanshu Biotechnology Co., Ltd.).

**Supplementary Table 2 List of phytochemicals identified in Amomum tsao-ko using ultra-high-performance liquid chromatography coupled with tandem mass spectrometry (UHPLC-MS/MS).**

| **No** | **Metabolite name** | **Formula** | **Mode** | **m/z** | **Mass Error (ppm)** | **Rt (Min)** | **Adducts** | **class** |
| --- | --- | --- | --- | --- | --- | --- | --- | --- |
| 1 | Oxyphyllenone A | C12H18O3 | pos | 252.1587606 | -5.871914469 | 2.209866667 | M+CH_3_CN+H | Organooxygen compounds |
| 2 | 3-Oxopentanedioic acid | C5H6O5 | neg | 191.0202717 | 3.755939111 | 0.916983333 | M-H, M+HCOO | Keto acids and derivatives |
| 3 | Obscuraminol F | C16H33NO | pos | 274.2733152 | -5.09079045 | 8.855816667 | M+H_2_O+H | Organonitrogen compounds |
| 4 | Procyanidin B1 | C30H26O12 | pos | 579.1482484 | -2.515541293 | 4.55415 | M+H | Flavonoids |
| 5 | Procyanidin C1 | C45H38O18 | neg | 865.2019202 | 3.904738805 | 4.636533333 | M-H | Flavonoids |
| 6 | 4-Methoxycinnamic acid | C10H10O3 | neg | 177.056251 | 2.994852131 | 6.77265 | M-H, M+Cl | Cinnamic acids and derivatives |
| 7 | Hyperoside | C21H20O12 | neg | 463.089972 | 3.818995917 | 5.788383333 | M+Cl, 2M-H, M-H | Flavonoids |
| 8 | Crotaleschenine | C16H23NO5 | pos | 328.1746351 | -4.488689376 | 1.664083333 | M+Na-H_2_O, M+H_2_O+H | Pyrrolizines |
| 9 | Heptadecanoic acid | C17H34O2 | pos | 288.2889847 | -2.668553239 | 9.3976 | M+NH_4_ | Fatty Acyls |
| 10 | Protocatechuic acid | C7H6O4 | neg | 153.0197554 | 2.747618428 | 3.4262 | M-H, M+Cl, 2M-H | Benzene and substituted derivatives |
| 11 | Isoquercitrin | C21H20O12 | pos | 465.1017322 | -2.198551367 | 5.8067 | M+H, M+K, M+Na | Flavonoids |
| 12 | 13,18-Dehydroglaucarubinone | C25H32O10 | pos | 457.1847088 | -2.00223212 | 6.788433333 | M+H-2H_2_O | Prenol lipids |
| 13 | Strophanthidin | C23H32O6 | pos | 445.2212612 | 2.653095749 | 8.9149 | M+H_2_O+Na | Steroids and steroid derivatives |
| 14 | Specneuzhenide | C31H42O17 | neg | 745.2535103 | -3.627894755 | 6.231216667 | M+CH_3_COO | Saccharolipids |
| 15 | 7-Hydroxy-5,8-dimethoxyflavanone | C17H16O5 | pos | 355.1166679 | 2.980025173 | 7.431316667 | M+CH_3_OH+Na | Flavonoids |
| 16 | L-GLUCOSE | C6H12O6 | pos | 145.049243 | -1.62262445 | 16.51335 | M+H-2H_2_O | Organooxygen compounds |
| 17 | Crobarbatine | C15H21NO5 | pos | 295.1645049 | -4.295830069 | 1.6226 | M+NH_4_-H_2_O | Pyrrolizines |
| 18 | Quercetin | C15H10O7 | pos | 303.0491555 | -2.561337749 | 5.8067 | M+H | Flavonoids |
| 19 | Protocatechualdehyde | C7H6O3 | neg | 137.0247797 | 2.62342129 | 4.6858 | M-H, M+Cl | Organooxygen compounds |
| 20 | 2,6,6-Trimethyl-2,4-cycloheptadien-1-one | C10H14O | pos | 169.1219467 | -6.118345355 | 6.6952 | M+H_2_O+H | Prenol lipids |
| 21 | Yadanzioside E | C32H44O16 | neg | 729.2581054 | -4.427942179 | 6.935433333 | M+HCOO | Prenol lipids |
| 22 | 8-Hydroxyoctanoic acid | C8H16O3 | pos | 224.1276207 | 8.596019179 | 1.947283333 | M+CH_3_CN+Na | Hydroxy acids and derivatives |
| 23 | Xanthoxyline | C10H12O4 | pos | 179.0698896 | -1.94315096 | 6.774533333 | M+H-H_2_O | Organooxygen compounds |
| 24 | Ophiopogonanone B | C18H18O5 | neg | 373.13038 | 3.681128882 | 7.345716667 | M+CH_3_COO | Homoisoflavonoids |
| 25 | Giffonin R | C19H16O3 | pos | 325.1426122 | -2.818576003 | 8.783216667 | M+CH3OH+H | Diarylheptanoids |
| 26 | 1,6,8-Trideoxyshanzhigenin | C10H14O3 | neg | 199.0982461 | 6.874599458 | 6.263516667 | M-H+H_2_O, M+CH_3_COOH+HCOO | Prenol lipids |
| 27 | Eupalinolide K | C20H26O6 | pos | 417.1897056 | 2.11801686 | 8.4664 | M+CH_3_OH+Na | Prenol lipids |
| 28 | Taxiresinol | C19H22O6 | neg | 391.1410982 | 3.639395855 | 5.693266667 | M+HCOO | Furanoid lignans |
| 29 | Dihydromorelloflavone | C30H22O11 | neg | 575.1216452 | 4.898049836 | 6.248416667 | M-H+H_2_O | Flavonoids |
| 30 | Scrophularoside A5 | C33H42O17 | neg | 727.2424713 | -3.419320742 | 7.128166667 | M-H+H_2_O | Cinnamic acids and derivatives |
| 31 | Dicrotaline | C14H19NO5 | pos | 281.1488582 | -4.498107355 | 1.632533333 | M+NH_4_-H_2_O, M+H | Pyrrolizines |
| 32 | L-Tyrosine | C9H11NO3 | pos | 182.0808267 | -1.894041191 | 1.0832 | M+H | Carboxylic acids and derivatives |
| 33 | Morusin | C25H24O6 | pos | 439.1741025 | -3.774651986 | 7.603966667 | M+H_2_O+H | Flavonoids |
| 34 | Cinobufagin | C26H34O6 | pos | 460.2681632 | -2.716079778 | 10.10376667 | M+NH_4_ | Steroids and steroid derivatives |
| 35 | Gliocladic acid | C14H22O4 | pos | 254.1744261 | -4.65517512 | 4.7112 | M+NH_4_-H_2_O | Prenol lipids |
| 36 | Lappaol F | C40H42O12 | neg | 731.2738891 | 4.987344468 | 7.86325 | M-H+H_2_O, M+CH_3_COO | 2-arylbenzofuran flavonoids |
| 37 | Caprylic acid | C8H16O2 | pos | 208.1327462 | 9.828285362 | 2.196683333 | M+CH_3_CN+Na | Fatty Acyls |
| 38 | 2,3-Dihydroxypterodontic acid | C15H22O4 | pos | 266.1743702 | -4.655115813 | 2.421983333 | M+NH_4_-H_2_O | Prenol lipids |
| 39 | Maleic acid | C4H4O4 | neg | 115.0039618 | 2.41068249 | 1.08705 | M-H | Carboxylic acids and derivatives |
| 40 | Caesalpine B | C23H30O7 | pos | 441.1901066 | 4.143125667 | 6.595533333 | M+Na | Prenol lipids |
| 41 | Kahweol | C20H26O3 | pos | 332.2211613 | -2.733712563 | 6.045383333 | M+NH_4_, M+K | Naphthofurans |
| 42 | 17-Hydroxyisolathyrol | C20H30O5 | pos | 350.2316602 | -4.180026389 | 5.637166667 | M+NH_4_-H_2_O | Prenol lipids |
| 43 | (4->2)-Abeo-16-hydroxycleroda-2,13-dien-15,16-olide-3-al | C20H28O4 | neg | 349.2031401 | 5.05937126 | 8.24405 | M-H+H_2_O | Prenol lipids |
| 44 | Methyl rosmarinate | C19H18O8 | neg | 433.1152704 | 3.482574524 | 4.6858 | M+CH_3_COO | Cinnamic acids and derivatives |
| 45 | Uvarigranol C | C23H24O7 | neg | 471.1678496 | 4.480155137 | 6.62915 | M+CH_3_COO | Benzene and substituted derivatives |
| 46 | Lucidone | C15H12O4 | neg | 273.0777216 | 5.712043783 | 5.629533333 | M-H, M-H+H_2_O | Cinnamic acids and derivatives |
| 47 | Dehydrohautriwaic acid | C20H26O4 | neg | 347.1874619 | 5.004983538 | 9.165966667 | M-H+H_2_O | Prenol lipids |
| 48 | Diosmin Impurity 5 | C30H38O15 | neg | 743.2373211 | -4.778049021 | 6.360316667 | M+CH_3_COOH+HCOO | Flavonoids |
| 49 | Marmin | C19H24O5 | neg | 363.1825569 | 5.36798544 | 7.9347 | M-H+CH_3_OH, M+HCOO | Coumarins and derivatives |
| 50 | Irisflorentin | C20H18O8 | pos | 409.0907681 | 3.573935403 | 6.449783333 | M+Na | Isoflavonoids |
| 51 | Ferulic acid | C10H10O4 | neg | 193.0512141 | 2.99752067 | 6.1096 | M-H, M+Cl | Cinnamic acids and derivatives |
| 52 | Arillanin A | C33H40O18 | neg | 741.22197 | -3.030075625 | 6.1096 | M-H+H_2_O | Cinnamic acids and derivatives |
| 53 | alpha-Mangostin | C24H26O6 | pos | 443.2054476 | -2.394435884 | 8.350433333 | M+H_2_O+H, M+CH_3_OH+H | Benzopyrans |
| 54 | L-Phenylalanine | C9H11NO2 | pos | 166.0859185 | -2.038883414 | 1.992533333 | M+H | Carboxylic acids and derivatives |
| 55 | 7,8,3',4'-tetrahydroxyflavanone | C15H12O6 | pos | 577.1327594 | -2.244726828 | 5.6931 | 2M+H | Flavonoids |
| 56 | 7-O-Methyleucomol | C18H18O6 | pos | 371.111564 | 2.80419483 | 7.065816667 | M+H_2_O+Na, 2M+H | Homoisoflavonoids |
| 57 | Naringin 4'-glucoside | C33H42O19 | neg | 759.2323691 | -3.179680466 | 6.184133333 | M-H+H_2_O | Flavonoids |
| 58 | Lychnopholic acid | C15H22O3 | pos | 314.1742121 | 4.067639861 | 5.22035 | M+CH_3_CN+Na | Prenol lipids |
| 59 | Kansuinine B | C38H42O14 | pos | 727.2366853 | 0.871222904 | 7.248566667 | M+H-H_2_O, M+Na-H_2_O | Carboxylic acids and derivatives |
| 60 | Vanillin | C8H8O3 | neg | 151.0405245 | 3.004337232 | 5.8682 | M-H | Phenols |
| 61 | Nepetin-7-glucoside | C22H22O12 | pos | 479.1172407 | -2.430168288 | 6.1419 | M+H | Flavonoids |
| 62 | Cucurbitacin D | C30H44O7 | pos | 516.3306288 | -3.631416889 | 11.04463333 | M+NH_4_-H_2_O | Steroids and steroid derivatives |
| 63 | Cnicin | C20H26O7 | pos | 343.153169 | -2.198114391 | 7.545883333 | M+H-2H_2_O | Prenol lipids |
| 64 | 16-Hydroxy-8(17),13-labdadien-15,16-olid-19-oic acid | C20H28O5 | neg | 365.1980761 | 4.888503598 | 7.4428 | M-H+H_2_O | Prenol lipids |
| 65 | Longicaulenone | C12H18O4 | pos | 268.1536544 | -5.548463676 | 2.0218 | M+CH_3_CN+H | Organooxygen compounds |
| 66 | 2-(2,4-dihydroxyphenyl)-3,5,7-trihydroxy-4H-chromen-4-one | C15H10O7 | neg | 301.036257 | 2.91619983 | 7.260083333 | M-H | Flavonoids |
| 67 | Lactupicrin | C23H22O7 | neg | 469.1519253 | 3.833438785 | 6.67965 | M+CH_3_COO | Phenols |
| 68 | 8alpha-Hydroxyhirsutinolide | C15H20O6 | pos | 296.1485007 | -4.34823742 | 2.36115 | M+NH_4_-H_2_O | Dihydrofurans |
| 69 | Chlorogenic Acid | C16H18O9 | pos | 437.1584852 | 7.068824697 | 7.931 | M+2CH_3_CN+H | Organooxygen compounds |
| 70 | Cinnamtannin B1 | C45H36O18 | pos | 865.19483 | -3.020993343 | 5.49495 | M+H | Flavonoids |
| 71 | Lysofungin | C27H49O12P | neg | 595.291103 | 3.715962014 | 10.6545 | M-H | Glycerophospholipids |
| 72 | L-Pyroglutamic acid | C5H7NO3 | neg | 128.0356712 | 2.747394815 | 0.98945 | M-H | Carboxylic acids and derivatives |
| 73 | Nicotinamide | C6H6N2O | pos | 123.0552303 | -0.483198442 | 1.452633333 | M+H | Pyridines and derivatives |
| 74 | Camellianin B | C27H30O14 | pos | 617.1276947 | 1.696644992 | 5.0593 | M+K | Flavonoids |
| 75 | Aromadendrin | C15H12O6 | neg | 333.0626659 | 3.741467858 | 5.407016667 | M+HCOO | Flavonoids |
| 76 | 1-(4-Hydroxybenzoyl)glucose | C13H16O8 | neg | 345.0837178 | 3.333530724 | 1.70395 | M+HCOO | Organooxygen compounds |
| 77 | 6-Feruloylcatalpol | C25H30O13 | neg | 597.1847011 | 4.199663824 | 5.709733333 | M+CH_3_COO | Cinnamic acids and derivatives |
| 78 | 3-O-Acetylpadmatin | C18H16O8 | neg | 377.0888074 | 4.415365728 | 4.818366667 | M+HCOO, M+CH_3_COO, M-H+H_2_O, M+Cl | Flavonoids |
| 79 | avicularin | C20H18O11 | neg | 433.079028 | 3.209132475 | 6.024683333 | M-H | Flavonoids |
| 80 | Tamarixetin | C16H12O7 | neg | 361.0576983 | 3.78280795 | 4.37125 | M+HCOO | Flavonoids |
| 81 | (+)-Junenol | C15H26O | pos | 205.1946732 | -1.818200866 | 8.9822 | M+H-H_2_O, M+H | Prenol lipids |
| 82 | Xanthatin | C15H18O3 | pos | 287.127022 | 4.581343924 | 7.603966667 | M+H_2_O+Na | Prenol lipids |
| 83 | Chromomoric acid B | C18H28O3 | neg | 351.218813 | 3.997989005 | 9.010183333 | M+CH_3_COO | Fatty Acyls |
| 84 | Paeonilactone B | C10H12O4 | neg | 195.0668781 | 3.037999025 | 5.658583333 | M-H, M+Cl | Benzofurans |
| 85 | Lobetyolin | C20H28O8 | neg | 441.1779815 | 3.441888365 | 5.709733333 | M+HCOO | Fatty Acyls |
| 86 | Hymatoxin D | C20H30O6S | neg | 457.1885063 | -4.026930414 | 7.968483333 | M+CH_3_COO | Prenol lipids |
| 87 | Lecithin | C42H80NO8P | neg | 816.5797414 | 4.997956771 | 11.61985 | M+CH_3_COO | Glycerophospholipids |
| 88 | Haploperoside E | C28H38O17 | neg | 645.2003641 | -5.04364588 | 6.67965 | M-H | Organooxygen compounds |
| 89 | Naringenin-7-O-beta-D-glucuronide | C21H20O11 | neg | 447.0948506 | 3.493881656 | 4.73025 | M-H | Flavonoids |
| 90 | 15-Hydroxy-7-oxodehydroabietic acid | C20H26O4 | pos | 330.2055804 | -4.024635012 | 6.1419 | M+NH_4_-H_2_O | Prenol lipids |
| 91 | Plumieride | C21H26O12 | pos | 435.1275762 | -2.120890422 | 4.393316667 | M+H-2H_2_O | Prenol lipids |
| 92 | Robustaside D | C21H22O10 | neg | 433.1152107 | 2.741771684 | 4.038583333 | M-H, M+Cl | Saccharolipids |
| 93 | Gingerglycolipid A | C33H56O14 | neg | 675.3623775 | 3.914176695 | 10.0884 | M-H, M+Cl, M+CH_3_COO | Glycerolipids |
| 94 | Morelloflavone | C30H20O11 | neg | 573.1060369 | 4.990958369 | 6.327983333 | M-H+H_2_O | Flavonoids |
| 95 | 12-Hydroxydodecanoic acid | C12H24O3 | pos | 280.189998 | 5.33547335 | 5.4247 | M+CH_3_CN+Na | Hydroxy acids and derivatives |
| 96 | Mulberroside C | C24H26O9 | neg | 489.1782601 | 4.753065909 | 5.20025 | M-H+CH_3_OH | 2-arylbenzofuran flavonoids |
| 97 | 5-Hydroxy-1-tetralone | C10H10O2 | pos | 181.0855145 | -5.954880405 | 5.718416667 | M+H_2_O+H | Tetralins |
| 98 | 1,8-Dihydroxy-p-menth-3-en-2-one | C10H16O3 | neg | 183.1032026 | 2.904187347 | 7.014733333 | M-H | Prenol lipids |
| 99 | Quercetin 7-rhamnoside | C21H20O11 | neg | 465.1052697 | 4.481653156 | 4.701166667 | M-H+H_2_O | Flavonoids |
| 100 | Caleurticolide 2-methylacrylate | C23H28O8 | pos | 474.2112182 | -3.70127483 | 5.718416667 | M+CH_3_CN+H | Prenol lipids |
| 101 | Chaetopenoid A | C24H30O6 | neg | 459.2048869 | 5.910626625 | 6.741916667 | M+HCOO | Prenol lipids |
| 102 | Obacunone | C26H30O7 | neg | 499.1991726 | 4.005428484 | 7.86325 | M-H, M+HCOO | Prenol lipids |
| 103 | 9-Aminocamptothecin | C20H17N3O4 | neg | 403.1410502 | 1.223125674 | 7.460283333 | M-H+CH_3_CN | Camptothecins |
| 104 | Neonuezhenide | C31H42O18 | neg | 761.2475425 | -4.801781932 | 5.963083333 | M+CH_3_COO | Saccharolipids |
| 105 | norbergenin | C13H14O9 | neg | 359.0631397 | 3.686213783 | 1.504066667 | M+HCOO | Benzene and substituted derivatives |
| 106 | Meridinol | C20H18O7 | pos | 411.1064914 | 2.535642876 | 6.882083333 | M+H_2_O+Na | Furanoid lignans |
| 107 | Rutin | C27H30O16 | pos | 611.1592808 | -2.262530851 | 5.612916667 | M+H | Flavonoids |
| 108 | Sclareol glycol | C16H30O2 | pos | 318.2419902 | 4.366400728 | 6.763716667 | M+CH_3_CN+Na | Organooxygen compounds |
| 109 | Lithospermic acid | C27H22O12 | neg | 537.1056552 | 3.355319988 | 9.200933333 | M-H | 2-arylbenzofuran flavonoids |
| 110 | Verproside | C22H26O13 | pos | 463.1246551 | 2.343131661 | 3.311933333 | M+H-2H_2_O | Organooxygen compounds |
| 111 | Methyl gallate | C8H8O5 | neg | 183.0304134 | 2.80643503 | 4.20415 | M-H | Benzene and substituted derivatives |
| 112 | 12-Hydroxyabietic acid | C20H30O3 | pos | 382.2365715 | 2.440584627 | 7.506766667 | M+CH_3_CN+Na | Prenol lipids |
| 113 | Nervonic acid | C24H46O2 | neg | 383.3541782 | 4.633472999 | 13.46705 | M-H+H_2_O | Fatty Acyls |
| 114 | Quercetin-3-O-glucose-6''-acetate | C23H22O13 | neg | 505.1004051 | 3.242033879 | 5.900533333 | M-H | Flavonoids |
| 115 | 6-O-Vanilloylsucrose | C20H28O14 | pos | 515.1361956 | -1.89095645 | 0.858466667 | M+Na | Tannins |
| 116 | Arglabin | C15H18O3 | pos | 247.132328 | -2.20556124 | 7.198466667 | M+H, M+H_2_O+H, M+H-H_2_O | Prenol lipids |
| 117 | MALTOTETRAOSE | C24H42O21 | neg | 665.2175297 | 4.424546951 | 15.22118333 | M-H, M+Cl, M+CH_3_COO | Organooxygen compounds |
| 118 | Taxifolin 7-rhamnoside | C21H22O11 | neg | 467.120826 | 4.253297745 | 4.324066667 | M-H+H_2_O, M+HCOOH+HCOO | Flavonoids |
| 119 | N-(1-Carboxy-2-phenylethyl)glutamine | C14H18N2O5 | pos | 367.1489333 | 2.929449695 | 2.123966667 | M+CH_3_OH+Na+H_2_O | Carboxylic acids and derivatives |
| 120 | 4,5-Dihydroblumenol A | C13H22O3 | pos | 268.1899677 | -5.867074032 | 4.55415 | M+CH_3_CN+H | Prenol lipids |
| 121 | (R)-3-Hydroxybutanoic acid | C4H8O3 | pos | 104.0708579 | -2.750735211 | 0.841666667 | M+NH_4_-H_2_O | Hydroxy acids and derivatives |
| 122 | Erythroskyrin | C26H33NO6 | pos | 496.2309942 | -0.205593313 | 4.585166667 | M+H_2_O+Na | Furofurans |
| 123 | 3-(Hydroxymethyl)-2-penten-5-olide | C6H8O3 | neg | 187.0617682 | 4.873983273 | 4.125116667 | M+CH_3_COO | Pyrans |
| 124 | 3,10-Dihydroxy-5,11-dielmenthadiene-4,9-dione | C20H28O4 | pos | 332.2212024 | -4.084749313 | 6.814783333 | M+NH_4_-H_2_O, M+Na-H_2_O | Prenol lipids |
| 125 | Lotaustralin | C11H19NO6 | pos | 325.1386072 | 4.097096509 | 5.186 | M+CH_3_CN+Na | Organooxygen compounds |
| 126 | Dodonolide | C20H24O3 | neg | 329.1768319 | 5.084505131 | 9.2357 | M-H+H_2_O | Dihydrofurans |
| 127 | 12-Hydroxyjasmonic acid | C12H18O4 | pos | 191.1062498 | -1.797070192 | 6.33525 | M+H-2H_2_O, M+Na | Fatty Acyls |
| 128 | Stearidonic Acid | C18H28O2 | neg | 293.213092 | 5.292592863 | 10.85655 | M-H+H_2_O | Fatty Acyls |
| 129 | Garcinoic acid | C27H38O4 | pos | 490.2916599 | -3.870275846 | 9.35265 | M+CH_3_CN+Na | Prenol lipids |
| 130 | Ethyl gallate | C9H10O5 | neg | 197.0461048 | 2.816239178 | 1.599783333 | M-H | Benzene and substituted derivatives |
| 131 | Auranticin A | C24H24O8 | neg | 485.1471695 | 4.207191754 | 6.1096 | M-H+H_2_O, M+HCOO | Depsides and depsidones |
| 132 | Methylswertianin | C15H12O6 | pos | 307.0804236 | -4.736723263 | 4.860833333 | M+H_2_O+H | Benzopyrans |
| 133 | Pueroside C | C24H26O10 | neg | 579.1740442 | 4.528362072 | 5.834183333 | M+Cl, M+CH_3_COOH+HCOO | Organooxygen compounds |
| 134 | coreopsin | C21H22O10 | pos | 453.1379603 | -4.000333064 | 3.115966667 | M+H_2_O+H | Flavonoids |
| 135 | Linocinnamarin | C16H20O8 | pos | 340.1382526 | -4.013431425 | 1.092083333 | M+NH_4_-H_2_O | Organooxygen compounds |
| 136 | 4-Hydroxymethylphenol 1-O-rhamnoside | C13H18O6 | neg | 269.1038577 | 2.946496099 | 5.10785 | M-H, M-H+H_2_O | Organooxygen compounds |
| 137 | Borreriagenin | C10H14O5 | neg | 213.0775459 | 3.264291342 | 4.790533333 | M-H | Lactones |
| 138 | 1-Methylinosine | C11H14N4O5 | neg | 341.109952 | -0.948388687 | 5.21685 | M+CH_3_COO | Purine nucleosides |
| 139 | Paeonol | C9H10O3 | neg | 211.0618737 | 4.091843869 | 4.73025 | M+HCOO | Organooxygen compounds |
| 140 | Loureiriol | C16H14O6 | neg | 347.0781938 | 3.163499814 | 4.9429 | M+HCOO | Homoisoflavonoids |
| 141 | Aromadendrin 4'-glucoside | C21H22O11 | neg | 449.1103809 | 3.212007222 | 4.305416667 | M-H | Flavonoids |
| 142 | Isorhamnetin-3-O-nehesperidine | C28H32O16 | neg | 623.1641023 | 3.755133458 | 5.963083333 | M-H | Flavonoids |
| 143 | Abscisic Acid | C15H20O4 | pos | 264.1586884 | -4.810764361 | 4.860833333 | M+NH_4_-H_2_O | Prenol lipids |
| 144 | Quercetin 3-O-Beta-D-Glucuronide | C21H18O13 | pos | 551.1018208 | 1.213449174 | 5.898666667 | M+CH_3_OH+Na+H_2_O | Flavonoids |
| 145 | Regaloside C | C18H24O11 | neg | 451.1052157 | 9.498910416 | 6.184133333 | M+Cl, M+CH_3_COOH+HCOO | Glycerolipids |
| 146 | Endomorphin-1 | C34H38N6O5 | neg | 701.2994112 | 8.787382417 | 7.409816667 | M+HCOOH+HCOO | Carboxylic acids and derivatives |
| 147 | trans-4-Hydroxycyclohexanecarboxylic Acid | C7H12O3 | neg | 189.0774834 | 4.436204218 | 5.35645 | M+HCOO | Organooxygen compounds |
| 148 | Pendulone | C17H16O6 | neg | 361.0940588 | 3.703157673 | 5.467866667 | M+HCOO | Isoflavonoids |
| 149 | Norpterosin B | C13H16O2 | pos | 187.1113421 | -1.956588344 | 7.09045 | M+H-H_2_O, M+NH_4_ | Indanes |
| 150 | Aigialomycin D | C18H22O6 | pos | 357.1323622 | 4.49832606 | 8.0064 | M+Na | Macrolides and analogues |
| 151 | Alpinin A | C20H24O7 | pos | 359.1480029 | -2.424490205 | 6.9161 | M+H-H_2_O | Diarylheptanoids |
| 152 | Piptocarphin F | C21H28O8 | pos | 472.1952995 | 1.425043728 | 7.931 | M+CH_3_CN+Na | Dihydrofurans |
| 153 | Odontoside | C20H22O11 | pos | 403.1037902 | 3.267763803 | 3.447716667 | M+H-2H_2_O | Organooxygen compounds |
| 154 | Demethylcarolignan E | C39H40O13 | neg | 715.2331557 | -9.018062374 | 4.884033333 | M-H |  |
| 155 | Choline glycerophosphate | C8H20NO6P | pos | 296.0652885 | -2.697614868 | 0.7762 | M+K | Glycerophospholipids |
| 156 | Toxin T2 tetrol | C15H22O6 | pos | 316.175962 | 1.670379797 | 4.812766667 | M+NH_4_ | Prenol lipids |
| 157 | trans-4-Hydroxy-2-nonenoic acid | C9H16O3 | neg | 217.1087789 | 3.686859318 | 5.646133333 | M+HCOO | Hydroxy acids and derivatives |
| 158 | 7-Methoxycoumarin | C10H8O3 | neg | 221.0462336 | 3.916226083 | 5.168133333 | M-H, M+HCOO | Coumarins and derivatives |
| 159 | Breyniaionoside A | C19H32O9 | neg | 509.2254759 | 3.818150475 | 4.189033333 | M+CH_3_COOH+HCOO | Fatty Acyls |
| 160 | Lycofawcine | C18H29NO4 | pos | 364.210758 | 2.474039362 | 5.626983333 | M+H_2_O+Na | Prenol lipids |
| 161 | Volkensiflavone | C30H20O10 | pos | 573.1378133 | -2.452723565 | 6.22575 | M+CH_3_OH+H | Flavonoids |
| 162 | Phloretin 3',5'-Di-C-glucoside | C27H34O15 | neg | 629.2052662 | -4.855034435 | 6.885133333 | M-H+CH_3_OH | Linear 1,3-diarylpropanoids |
| 163 | Salvianolic acid A | C26H22O10 | neg | 493.1158429 | 3.688168214 | 6.126783333 | M-H | Stilbenes |
| 164 | Foliachinenoside C | C28H38O14 | neg | 657.2366531 | -5.521056399 | 7.764 | M+CH_3_COO | Lignan glycosides |
| 165 | Juglanin | C20H18O10 | neg | 449.1105704 | 5.198916088 | 4.537483333 | M-H+CH_3_OH | Flavonoids |
| 166 | ent-11alpha-Hydroxyabieta-8(14),13(15)-dien-16,12alpha-olide | C20H28O3 | pos | 334.236812 | -2.714227487 | 4.910816667 | M+NH_4_ | Prenol lipids |
| 167 | Psoromic acid | C18H14O8 | neg | 417.0840705 | 3.918387348 | 5.820916667 | M+CH_3_COO | Depsides and depsidones |
| 168 | Aloenin B | C34H38O17 | neg | 753.185572 | 7.338942592 | 6.024683333 | M+Cl | Organooxygen compounds |
| 169 | Preisocalamendiol | C15H24O | pos | 203.1790662 | -1.63941441 | 6.608233333 | M+H-H_2_O | Prenol lipids |
| 170 | Heudelotinone | C18H20O2 | pos | 286.1791744 | -3.658592696 | 4.98745 | M+NH_4_ | Phenols |
| 171 | 6'-Hydroxy-3,4,2',3',4'-pentamethoxychalcone | C20H22O7 | neg | 405.1566891 | 4.640166178 | 6.248416667 | M-H+CH_3_OH, M+HCOO | Linear 1,3-diarylpropanoids |
| 172 | Lecocarpinolide H | C15H18O5 | neg | 323.1145738 | 3.416378136 | 6.610716667 | M+HCOO | Prenol lipids |
| 173 | Thunberginol C | C15H12O5 | neg | 331.0833302 | 3.883991948 | 4.305416667 | M+CH_3_COO | Benzene and substituted derivatives |
| 174 | Tecomelloside | C24H30O13 | pos | 631.2154938 | 7.512339575 | 7.545883333 | M+2CH_3_CN+Na | Tannins |
| 175 | Bruceoside A | C32H42O16 | neg | 727.242505 | -4.368273545 | 6.197933333 | M+HCOO | Prenol lipids |
| 176 | 1-Dehydroperuvinine | C15H18O4 | pos | 263.1271837 | -2.295954875 | 5.704716667 | M+H, M+H_2_O+H, M+H-H_2_O | Prenol lipids |
| 177 | ent-14,16-Epoxy-8-pimarene-3,15-diol | C20H32O3 | pos | 353.2676751 | -3.000871783 | 10.95301667 | M+CH_3_OH+H | Steroids and steroid derivatives |
| 178 | Sugiol | C20H28O2 | pos | 300.2314534 | -4.252915863 | 12.5462 | M+NH_4_-H_2_O | Prenol lipids |
| 179 | 2,7-Dihydrohomoerysotrine | C20H27NO3 | pos | 348.2160634 | -4.344772348 | 5.22035 | M+H_2_O+H | Erythrina alkaloids |
| 180 | Ligustilide | C12H14O2 | pos | 191.1062522 | -2.124894215 | 6.132883333 | M+H | Isobenzofurans |
| 181 | Usnic Acid | C18H16O7 | neg | 389.089063 | 3.662257567 | 4.790533333 | M+HCOO | Benzene and substituted derivatives |
| 182 | 2-Hydroxy-7-O-methylscillascillin | C18H14O7 | neg | 341.0677273 | 3.07235421 | 6.197933333 | M-H, M+Cl | Isoflavonoids |
| 183 | 6''-O-(3-Hydroxy-3-methylglutaroyl)hyperin | C27H28O16 | neg | 625.1435484 | 5.119917863 | 4.636533333 | M-H+H_2_O | Flavonoids |
| 184 | Uridine | C9H12N2O6 | neg | 243.0628337 | 2.351936907 | 1.08705 | M-H, M+Cl, M+CH_3_COO | Pyrimidine nucleosides |
| 185 | Labdanolic acid | C20H36O3 | pos | 388.2834892 | 2.295137095 | 8.549816667 | M+CH_3_CN+Na | Prenol lipids |
| 186 | 4-Hydroxy-3-methoxymandelic acid | C9H10O5 | pos | 419.096251 | 3.492681221 | 5.794066667 | 2M+Na | Phenols |
| 187 | 1-Oleoyl-sn-glycero-3-phosphocholine | C26H52NO7P | neg | 580.3640831 | 4.111312563 | 11.48925 | M+Cl, M+CH_3_COO | Glycerophospholipids |
| 188 | alpha-L-Rhamnopyranose | C6H12O5 | neg | 181.0723085 | 6.917662336 | 0.80845 | M-H+H_2_O, M+CH_3_COOH+HCOO | Organooxygen compounds |
| 189 | Cyclo(Tyr-Val) | C14H18N2O3 | pos | 304.1648413 | -4.963888184 | 4.1585 | M+K, M+CH_3_CN+H | Carboxylic acids and derivatives |
| 190 | Goniothalamin | C13H12O2 | neg | 245.0826692 | 3.696416385 | 8.108816667 | M+HCOO | Benzene and substituted derivatives |
| 191 | Baicalin | C21H18O11 | pos | 447.0911056 | -2.425949854 | 6.449783333 | M+H | Flavonoids |
| 192 | Obscurolide A1 | C15H17NO5 | pos | 310.1276822 | -4.776368924 | 2.123966667 | M+H_2_O+H | Benzene and substituted derivatives |
| 193 | 4'-O-Methyl-8-prenylnaringenin | C21H22O5 | neg | 385.1669198 | 5.071409041 | 8.209466667 | M-H+CH_3_OH | Flavonoids |
| 194 | Schisanhenol | C23H30O6 | pos | 443.2053205 | 1.943954185 | 5.732433333 | M+H_2_O+Na | Tannins |
| 195 | Euparone | C12H10O4 | neg | 235.0618923 | 5.886138986 | 4.40465 | M-H+H_2_O, M+CH_3_COOH+HCOO, M+HCOOH+HCOO | Benzofurans |
| 196 | 5-Epilithospermoside | C14H19NO8 | pos | 347.1439569 | -2.841712222 | 1.380666667 | M+H-H_2_O, M+NH_4_ | Organooxygen compounds |
| 197 | Delta-Caesalpin | C20H30O6 | pos | 366.226578 | -3.988672406 | 6.7305 | M+NH_4_-H_2_O | Prenol lipids |
| 198 | Aesculetin (Cichorigenin | C9H6O4 | neg | 209.0461747 | 6.549490211 | 4.9429 | M-H+CH_3_OH | Coumarins and derivatives |
| 199 | Betavulgaroside IV | C41H62O15 | neg | 825.4315166 | 5.343971458 | 6.8531 | M-H+CH_3_OH | Prenol lipids |
| 200 | Picrocrocin | C16H26O7 | pos | 330.1900436 | -4.875114264 | 4.860833333 | M+NH_4_-H_2_O | Organooxygen compounds |
| 201 | Periplanetin | C13H16O7 | neg | 329.0887618 | 3.375374882 | 1.4025 | M+HCOO | Organooxygen compounds |
| 202 | Moluccanin | C20H18O8 | neg | 385.0940089 | 2.895051219 | 4.790533333 | M-H | Coumarinolignans |
| 203 | Isoboonein | C9H14O3 | pos | 135.0802412 | -1.176628944 | 10.10376667 | M+H-2H_2_O | Prenol lipids |
| 204 | Salviaplebeiaside | C23H26O10 | pos | 467.1327391 | 3.336650563 | 6.524416667 | M+Na-H_2_O, M+H_2_O+H | Organooxygen compounds |
| 205 | Dehydroglyasperin C | C21H22O5 | neg | 413.1617808 | 3.549420269 | 7.899916667 | M+CH_3_COO | Isoflavonoids |
| 206 | Sphingofungin B | C20H39NO6 | pos | 354.2628392 | -2.686635887 | 5.14055 | M+H-2H_2_O | Fatty Acyls |
| 207 | Noroxyhydrastinine | C10H9NO3 | neg | 236.057109 | 3.485215368 | 2.4464 | M+HCOO | Isoquinolines and derivatives |
| 208 | Paullinic acid | C20H38O2 | pos | 310.3096382 | -4.325982253 | 14.80373333 | M+NH_4_-H_2_O | Fatty Acyls |
| 209 | Nobiletin | C21H22O8 | pos | 425.1220402 | 3.361615368 | 6.82545 | M+Na | Flavonoids |
| 210 | 1-Linoleoyl-sn-glycero-3-phosphorylcholine | C26H50NO7P | neg | 578.3485058 | 4.267538382 | 10.77301667 | M+Cl, M+CH_3_COO | Glycerophospholipids |
| 211 | Oleuropeinic acid | C25H30O15 | neg | 605.1324012 | 7.944500538 | 5.0308 | M+Cl | Prenol lipids |
| 212 | Picraquassioside B | C19H24O11 | pos | 433.1118196 | 3.171932708 | 4.585166667 | M+Na-H_2_O | Organooxygen compounds |
| 213 | 4-(Dimethylamino)cinnamic acid | C11H13NO2 | pos | 210.1120429 | -5.158416451 | 1.813483333 | M+H_2_O+H | Cinnamic acids and derivatives |
| 214 | 5-Dehydroxyparatocarpin K | C20H18O4 | neg | 353.1405864 | 5.207283092 | 8.906866667 | M-H+CH_3_OH | Flavonoids |
| 215 | 14-Deoxy-17-hydroxyandrographolide | C20H32O5 | neg | 351.2188532 | 3.280525592 | 8.605466667 | M-H | Prenol lipids |
| 216 | Opuntiol | C7H8O4 | neg | 155.0354393 | 2.928813852 | 3.857733333 | M-H | Pyrans |
| 217 | S-Adenosyl-DL-Methionine | C15H22N6O5S | neg | 429.1567667 | 2.832838024 | 8.52085 | M-H+CH_3_OH | 5'-deoxyribonucleosides |
| 218 | Scutebarbatine Z | C26H33NO5 | pos | 480.2363258 | 0.347420494 | 5.344466667 | M+H_2_O+Na | Prenol lipids |
| 219 | Neoruscogenin | C27H40O4 | pos | 492.3073627 | -3.728864197 | 9.812316667 | M+CH_3_CN+Na | Prenol lipids |
| 220 | 12a-Hydroxydalpanol | C23H24O8 | pos | 469.148042 | 1.458140224 | 5.971266667 | M+H_2_O+Na | Isoflavonoids |
| 221 | Ethyl arachidonate | C22H36O2 | pos | 374.3043097 | -4.877571034 | 10.84516667 | M+CH_3_CN+H | Fatty Acyls |
| 222 | Dihydrojasmone | C11H18O | neg | 211.1346325 | 4.016800949 | 10.50078333 | M-H, M+HCOO | Organooxygen compounds |
| 223 | trans-3-Oxo-alpha-ionol | C13H20O2 | pos | 226.1796399 | -2.476528604 | 7.970366667 | M+NH_4_ | Prenol lipids |
| 224 | Helveticoside | C29H42O9 | neg | 569.2469559 | -9.972804493 | 4.22135 | M+Cl | Steroids and steroid derivatives |
| 225 | 3''-p-Coumaroylprunin | C30H28O12 | pos | 621.1589731 | 1.028643599 | 5.14055 | M+H_2_O+Na | Flavonoids |
| 226 | Emodin | C15H10O5 | neg | 269.046292 | 2.758665618 | 10.31103333 | M-H | Anthracenes |
| 227 | Musellactone | C13H12O4 | neg | 231.0669672 | 2.950534202 | 7.095933333 | M-H | Naphthofurans |
| 228 | copyranosyl ester | C26H38O10 | neg | 527.2515259 | 4.563393491 | 6.1096 | M-H+H_2_O | Prenol lipids |
| 229 | Peroxydehydrotumulosic acid | C31H46O6 | neg | 554.3482252 | 0.167244659 | 11.17753333 | M-H+CH_3_CN | Prenol lipids |
| 230 | Mono-O-methylcurvulinic acid | C11H12O5 | neg | 269.0674234 | 3.347033284 | 2.023233333 | M+HCOO | Organooxygen compounds |
| 231 | Sterebin A | C18H30O4 | neg | 309.2080847 | 3.067785972 | 9.587283333 | M-H | Prenol lipids |
| 232 | 3-Feruloyl-4-caffeoylquinic acid | C26H26O12 | pos | 581.1846916 | -4.40379826 | 5.460033333 | M+H_2_O+CH_3_OH+H | Organooxygen compounds |
| 233 | Pramanicin | C19H31NO6 | pos | 420.2580894 | -4.464325261 | 6.132883333 | M+H_2_O+CH_3_OH+H | Organooxygen compounds |
| 234 | Norepinephrine | C8H11NO3 | pos | 169.0968005 | -5.280160959 | 1.0832 | M+NH_4_-H_2_O | Phenols |
| 235 | Sucrose | C12H22O11 | neg | 401.1311803 | 3.416211026 | 5.045833333 | M+CH_3_COO | Organooxygen compounds |
| 236 | Isosalvianolic acid C | C26H20O10 | pos | 556.1220699 | 0.250119706 | 3.45765 | M+CH_3_CN+Na | Benzoxepines |
| 237 | Helenalin | C15H18O4 | neg | 523.2357075 | 3.749914146 | 8.24405 | 2M-H | Prenol lipids |
| 238 | Emodin-8-O-Beta-D-glucopyranoside | C21H20O10 | neg | 431.0998655 | 3.46006736 | 7.19505 | M-H | Anthracenes |
| 239 | Cornuside | C24H30O14 | pos | 647.210185 | 6.878618973 | 6.132883333 | M+2CH_3_CN+Na | Prenol lipids |
| 240 | Rubroside G | C33H38Cl2N2O9 | neg | 711.1597589 | -7.510613158 | 4.9429 | M+Cl | Organooxygen compounds |
| 241 | Homononactinic acid | C11H20O4 | neg | 215.1294747 | 2.739253551 | 8.89085 | M-H | Oxolanes |
| 242 | Methyl lucidenate D | C30H40O8 | neg | 527.2669815 | 3.671851325 | 8.503833333 | M-H | Prenol lipids |
| 243 | (+)-Mellein | C10H10O3 | pos | 143.0501452 | 5.638219612 | 0.64545 | M+H-2H_2_O | Benzopyrans |
| 244 | Calcium pantothenate |  | neg | 218.1039793 | 2.197451495 | 2.765066667 | M+ | Carboxylic acids and derivatives |
| 245 | Quercetol C | C22H24O5 | neg | 413.1618512 | 3.469448165 | 8.176666667 | M+HCOO | Flavonoids |
| 246 | Salvianolic acid C | C26H20O10 | neg | 537.1058588 | 4.088473827 | 6.126783333 | M+HCOO | 2-arylbenzofuran flavonoids |
| 247 | (-)-Catechin gallate | C22H18O10 | pos | 425.0856267 | -2.446969293 | 5.704716667 | M+H-H_2_O | Flavonoids |
| 248 | Engeletin | C21H22O10 | neg | 451.1260824 | 4.803939825 | 5.834183333 | M-H+H_2_O | Flavonoids |
| 249 | Colladonin angelate | C29H36O5 | neg | 509.2562431 | 3.810139199 | 7.764 | M+HCOO | Coumarins and derivatives |
| 250 | Glycyroside | C27H30O13 | neg | 579.1745128 | 5.642025054 | 5.453283333 | M-H+H_2_O | Isoflavonoids |
| 251 | Crotonionoside C | C24H42O12 | pos | 540.3054954 | 7.741486419 | 10.10376667 | M+NH_4_ | Prenol lipids |
| 252 | Gaultherin | C19H26O12 | neg | 505.1578187 | 3.569282828 | 4.125116667 | M+CH_3_COO | Organooxygen compounds |
| 253 | Galeopsin | C22H32O5 | pos | 376.2472597 | -4.025233229 | 7.603966667 | M+NH_4_-H_2_O | Prenol lipids |
| 254 | Kushenol X | C25H28O7 | pos | 440.2046994 | -5.915383965 | 7.959583333 | M+NH_4_-H_2_O | Flavonoids |
| 255 | Preglabridin | C20H22O4 | neg | 385.1669574 | 4.133624299 | 9.010183333 | M+CH_3_COO | Isoflavonoids |
| 256 | Pectolinarigenin | C17H14O6 | neg | 359.0783599 | 3.571449052 | 5.804366667 | M+HCOO | Flavonoids |
| 257 | 2-C-Methyl-D-erythrono-1,4-lactone | C5H8O4 | neg | 177.0409709 | 3.878992606 | 1.074516667 | M+HCOO | Lactones |
| 258 | Antibiotic PF 1018 | C28H35NO3 | neg | 473.2840787 | 8.504687224 | 13.46705 | M-H+CH_3_CN | Pyrrolizidines |
| 259 | Saprirearine | C20H24O2 | neg | 313.1818939 | 5.280152217 | 9.286983333 | M-H+H_2_O | Tetralins |
| 260 | 4(15),5,10(14)-Germacratrien-1-ol | C15H24O | neg | 265.1816722 | 3.436971657 | 9.794666667 | M+HCOO | Prenol lipids |
| 261 | Alpha-Linolenic acid | C18H30O2 | pos | 296.2576912 | -2.568077814 | 11.3495 | M+NH_4_ | Fatty Acyls |
| 262 | ()-Naringenin | C15H12O5 | neg | 317.0675578 | 3.250522151 | 4.37125 | M+HCOO | Flavonoids |
| 263 | Desmethylrocaglamide | C28H29NO7 | pos | 524.226816 | -2.193538872 | 6.118216667 | M+CH_3_OH+H | Benzofurans |
| 264 | 8-Methoxybonducellin | C18H16O5 | neg | 371.1147009 | 3.612003026 | 6.126783333 | M+CH_3_COO | Homoisoflavonoids |
| 265 | Rauvotetraphylline C | C28H33N2O7 | pos | 514.2057118 | -3.268891259 | 5.448933333 | M+Na-H_2_O | Macroline alkaloids |
| 266 | 4-Methyl-6,7-dihydroxycoumarin | C10H8O4 | neg | 237.0411187 | 3.436524168 | 2.781066667 | M-H, M+HCOO | Coumarins and derivatives |
| 267 | Phloyoside II | C17H25ClO12 | pos | 498.1392796 | 3.125742294 | 8.298783333 | M+CH_3_CN+H | Prenol lipids |
| 268 | 3-Hydroxymethylenetanshinquinone | C18H14O4 | neg | 339.0884199 | 3.437579755 | 7.712 | M+HCOO | Prenol lipids |
| 269 | Fraxinellone | C14H16O3 | pos | 233.1167022 | -2.234261123 | 5.718416667 | M+H-H_2_O, M+H | Isobenzofurans |
| 270 | Harmaline | C13H14N2O | pos | 233.1279213 | -5.098999737 | 4.860833333 | M+H_2_O+H | Harmala alkaloids |
| 271 | Gibepyrone D | C10H10O4 | neg | 387.1097798 | 3.191430306 | 7.144683333 | M-H+CH_3_OH, 2M-H | Pyrans |
| 272 | Pantoyllactone glucoside | C12H20O8 | neg | 383.1207562 | 4.308718434 | 1.1002 | M+HCOOH+HCOO | Fatty Acyls |
| 273 | Robustaside G | C30H28O13 | neg | 595.1475083 | 3.008864896 | 4.805566667 | M-H | Saccharolipids |
| 274 | ()-8-Gingerol | C19H30O4 | neg | 367.2137228 | 3.455338403 | 6.52675 | M+HCOO | Phenols |
| 275 | 8,9-Didehydro-7-hydroxydolichodial | C10H12O3 | neg | 225.07755 | 3.919121672 | 4.7747 | M+HCOO | Prenol lipids |
| 276 | Catechin 3-rhamnoside | C21H24O10 | pos | 441.1168831 | 3.06348627 | 5.1186 | M+Na-H_2_O | Flavonoids |
| 277 | Virginiaebutanolide A | C12H22O4 | neg | 229.145185 | 2.834044428 | 9.062533333 | M-H | Fatty Acyls |
| 278 | Securiterpenoside | C11H18O8 | neg | 337.1150346 | 3.837012857 | 1.061183333 | M+CH_3_COO | Saccharolipids |
| 279 | 5-(3-Hydroxypropyl)-7-methoxybenzofuran | C12H14O3 | neg | 251.0931489 | 3.176014192 | 6.248416667 | M+HCOO | Benzofurans |
| 280 | Hexanorcucurbitacin F | C24H36O5 | pos | 404.2785243 | -3.834114514 | 8.506983333 | M+NH_4_-H_2_O | Steroids and steroid derivatives |
| 281 | Maclurin | C13H10O6 | neg | 293.0675596 | 5.424931612 | 1.55015 | M-H+CH_3_OH | Benzene and substituted derivatives |
| 282 | Pierisformoside B | C26H42O8 | neg | 527.2878195 | 3.423601896 | 10.92211667 | M+HCOO | Organooxygen compounds |
| 283 | Limocitrin-3-O-rutinoside | C29H34O17 | neg | 689.1540844 | 7.770810148 | 5.931566667 | M+Cl | Flavonoids |
| 284 | LysoPC(14:0/0:0) | C22H46NO7P | pos | 468.3074213 | -2.235375796 | 10.0463 | M+H | Glycerophospholipids |
| 285 | Rhamnocitrin | C16H12O6 | neg | 345.0626433 | 3.516553717 | 5.123833333 | M-H+CH_3_OH, M+HCOO | Flavonoids |
| 286 | Unedone | C13H20O4 | pos | 205.1218986 | -1.697082144 | 6.9161 | M+H-2H_2_O | Organooxygen compounds |
| 287 | Myricanone | C21H24O5 | neg | 387.182965 | 6.152052474 | 8.19345 | M-H+CH_3_OH | Diarylheptanoids |
| 288 | Matairesinol | C20H22O6 | neg | 393.1122384 | 3.347048517 | 7.579716667 | M+Cl | Furanoid lignans |
| 289 | Syringic acid | C9H10O5 | neg | 197.0461431 | 3.010067231 | 4.091583333 | M-H | Benzene and substituted derivatives |
| 290 | Isoshinanolone | C11H12O3 | neg | 237.0775446 | 3.646102738 | 5.094116667 | M+HCOO | Tetralins |
| 291 | Linolenic acid | C18H30O2 | pos | 278.2471762 | -4.327369508 | 13.26626667 | M+NH_4_-H_2_O | Fatty Acyls |
| 292 | Evernic acid | C17H16O7 | neg | 331.0833185 | 2.987347067 | 3.129666667 | M-H | Depsides and depsidones |
| 293 | Astragalin | C21H20O11 | neg | 447.0950583 | 3.957367152 | 6.126783333 | M-H | Flavonoids |
| 294 | Citropten | C11H10O4 | neg | 251.0569178 | 3.925968937 | 5.629533333 | M+HCOO | Coumarins and derivatives |
| 295 | Isorhamnetin 3-glucuronide | C22H20O13 | neg | 551.1063053 | 4.297086733 | 4.9138 | M+CH_3_COO | Flavonoids |
| 296 | Apigenin 7-O-(6''-O-malonylglucoside) | C24H22O13 | neg | 535.1108837 | 4.136048655 | 5.35645 | M-H+H_2_O | Flavonoids |
| 297 | Alnuside A | C24H30O9 | neg | 461.1832618 | 3.365931459 | 8.019766667 | M-H | Diarylheptanoids |
| 298 | 3,3'-Di-O-methylellagic acid | C16H10O8 | pos | 331.0465536 | 5.18077982 | 4.860833333 | M+H | Tannins |
| 299 | Genistein | C15H10O5 | pos | 271.0593619 | -2.732651496 | 4.963166667 | M+H | Isoflavonoids |
| 300 | Boschnaloside | C16H24O8 | pos | 309.1324462 | -2.378545161 | 7.282266667 | M+H-2H_2_O | Prenol lipids |
| 301 | Uvarigranol B | C23H22O8 | neg | 485.1472629 | 4.68224477 | 5.248583333 | M+CH_3_COO | Benzene and substituted derivatives |
| 302 | Isofraxinellone | C14H16O3 | neg | 249.1139628 | 5.680249439 | 8.52085 | M-H+H_2_O | Isobenzofurans |
| 303 | 12-Hydroxymyricanone | C21H24O6 | neg | 431.1724697 | 3.711430735 | 7.8816 | M+CH_3_COO | Diarylheptanoids |
| 304 | Anisic aldehyde | C8H8O2 | pos | 169.085594 | -2.401071974 | 9.29225 | M+CH_3_OH+H | Benzene and substituted derivatives |
| 305 | Trijugin C | C22H34O6 | pos | 394.2578059 | -3.888349734 | 7.603966667 | M+NH_4_-H_2_O | Prenol lipids |
| 306 | Artemyriantholide D | C30H36O6 | neg | 509.2564308 | 5.163824727 | 8.108816667 | M-H+H_2_O | Prenol lipids |
| 307 | Lucidumoside C | C27H36O14 | pos | 549.192931 | -6.370802656 | 5.392416667 | M+H-2H_2_O | Prenol lipids |
| 308 | Neoagarobiose | C12H20O10 | pos | 365.104332 | -4.982420653 | 16.68275 | M+H_2_O+Na | Organooxygen compounds |
| 309 | Vitexilactone | C22H34O5 | pos | 378.2629065 | -4.012405371 | 8.047266667 | M+NH_4_-H_2_O | Prenol lipids |
| 310 | Lauroside A | C19H32O8 | neg | 479.2148893 | 3.841642981 | 4.125116667 | M+HCOOH+HCOO | Fatty Acyls |
| 311 | Pterolactam | C5H9NO2 | neg | 150.0326941 | -0.310520267 | 6.152966667 | M+Cl | Pyrrolidines |
| 312 | Lonfuranacid A | C12H20O5 | pos | 286.1642542 | -4.995571885 | 5.35975 | M+CH_3_CN+H | Fatty Acyls |
| 313 | Hispanolone | C20H30O3 | neg | 335.2238604 | 5.23418144 | 8.4684 | M-H+H_2_O | Prenol lipids |
| 314 | Lactobionic acid | C12H22O12 | neg | 417.1260702 | 3.194845467 | 0.8371 | M+CH_3_COO | Fatty Acyls |
| 315 | Laetisaric acid | C18H32O3 | pos | 360.2522865 | 2.841085568 | 7.545883333 | M+CH_3_CN+Na | Fatty Acyls |
| 316 | Stachyose | C24H42O21 | neg | 665.2176935 | 4.670453148 | 5.21685 | M-H | Organooxygen compounds |
| 317 | Carnosol | C20H26O4 | neg | 329.1768554 | 3.096799688 | 10.80876667 | M-H | Prenol lipids |
| 318 | (-)-Epiafzelechin 3-O-gallate | C22H18O9 | neg | 457.1158669 | 5.596767862 | 5.0149 | M-H+CH_3_OH | Flavonoids |
| 319 | Calyxanthone | C15H10O6 | neg | 345.0626412 | 3.856662207 | 5.4229 | M+CH_3_COO | Benzopyrans |
| 320 | Isorhamnetin | C16H12O7 | neg | 315.0521642 | 3.600191391 | 8.0555 | M-H | Flavonoids |
| 321 | Nepetoidin B | C17H14O6 | neg | 313.0727505 | 3.14822095 | 6.837583333 | M-H, M+CH_3_COO | Cinnamic acids and derivatives |
| 322 | Phloridzin | C21H24O10 | neg | 435.1311206 | 3.324745901 | 5.3285 | M-H | Flavonoids |
| 323 | trans-Norpterosin C | C13H16O3 | neg | 219.1033593 | 3.140872184 | 8.57035 | M-H | Indanes |
| 324 | Demethylwedelolactone | C15H8O7 | neg | 299.0205855 | 2.864354804 | 7.277216667 | M-H, M+HCOOH+HCOO | Isoflavonoids |
| 325 | Dasycarpol | C14H16O4 | neg | 293.1040845 | 4.133444301 | 6.059033333 | M+HCOO | Isobenzofurans |
| 326 | Caffeic acid | C9H8O4 | neg | 359.0781596 | 2.550967338 | 7.19505 | M-H+H_2_O, 2M-H | Cinnamic acids and derivatives |
| 327 | (15:2)-Anacardic acid | C22H32O3 | pos | 386.2678781 | -4.841998343 | 12.06938333 | M+CH_3_CN+H | Fatty Acyls |
| 328 | Dihydrokavain | C14H16O3 | neg | 277.1089571 | 3.501325085 | 5.978733333 | M+HCOO | Kavalactones |
| 329 | parthenolide | C15H20O3 | pos | 289.1427365 | 4.803696658 | 9.0497 | M+H_2_O+Na | Prenol lipids |
| 330 | 13,16,19-Docosatrienoic acid | C22H38O2 | pos | 334.3099057 | -3.214939647 | 14.20708333 | M+NH_4_-H_2_O | Fatty Acyls |
| 331 | 6-Gingerol | C17H26O4 | neg | 293.1767886 | 3.248592363 | 9.49875 | M-H | Phenols |
| 332 | Cholic acid | C24H40O5 | neg | 407.2815317 | 3.021731504 | 9.165966667 | M-H | Steroids and steroid derivatives |
| 333 | Robustaside A | C21H22O9 | pos | 418.1484089 | -4.241363834 | 4.963166667 | M+NH_4_-H_2_O | Organooxygen compounds |
| 334 | Wedelolactone | C16H10O7 | neg | 359.0420195 | 3.715619752 | 4.37125 | M+HCOO | Isoflavonoids |
| 335 | Cinchonain Ia | C24H20O9 | neg | 451.1055353 | 4.599529941 | 4.96715 | M-H | Flavonoids |
| 336 | Aloeresin D | C29H32O11 | pos | 574.226685 | -2.880953269 | 5.095933333 | M+NH_4_ | Organooxygen compounds |
| 337 | Azelaic acid | C9H16O4 | neg | 187.0982027 | 3.296539593 | 7.478966667 | M-H | Fatty Acyls |
| 338 | Tenuifoliside B | C30H36O17 | neg | 773.2119909 | -3.829357626 | 4.930866667 | M+CH_3_COOH+HCOO | Cinnamic acids and derivatives |
| 339 | Curcumenol | C15H22O2 | pos | 235.1686976 | -2.386236351 | 9.539 | M+H | Prenol lipids |
| 340 | Lasiodiplodin | C17H24O4 | pos | 310.2005169 | -2.628113021 | 4.51335 | M+NH_4_ | Macrolides and analogues |
| 341 | 3'-Demethylnobiletin | C20H20O8 | pos | 371.1116515 | -2.261917426 | 6.39625 | M+H-H_2_O | Flavonoids |
| 342 | 10-O-trans-p-Feruloylscandoside | C26H30O14 | neg | 611.1643739 | 4.62456721 | 4.58555 | M+HCOO | Prenol lipids |
| 343 | Deodarin | C32H26O14 | neg | 665.1537823 | 4.930784406 | 4.831933333 | M-H+CH_3_OH | Flavonoids |
| 344 | 8-methyl-8-azabicyclo[3.2.1]oct-3-yl hydroxy(phenyl)acetate | C16H21NO3 | pos | 276.1587537 | -2.421541247 | 5.035183333 | M+H | Tropane alkaloids |
| 345 | Adenosine | C10H13N5O4 | neg | 326.1115171 | 3.606238168 | 1.676216667 | M-H, M+Cl, M+CH_3_COO | Purine nucleosides |
| 346 | Anemonin | C10H8O4 | neg | 209.0461926 | 6.425136127 | 5.629533333 | M-H+H_2_O | Dihydrofurans |
| 347 | Boscialin | C13H22O3 | neg | 271.1559957 | 3.984444788 | 7.712 | M+HCOO | Prenol lipids |
| 348 | (+)-atechin 5-gallate | C22H18O10 | neg | 441.0839924 | 2.877285566 | 2.4464 | M-H | Flavonoids |
| 349 | Prunasin acid | C14H18O8 | pos | 332.1331481 | -2.69006035 | 1.0444 | M+NH_4_ | Organooxygen compounds |
| 350 | Anonamine | C19H28NO7+ | neg | 381.1777409 | -4.081646648 | 4.091583333 | M-H |  |
| 351 | Guanosine | C10H13N5O5 | neg | 282.0851025 | 2.509483213 | 1.08705 | M-H | Purine nucleosides |
| 352 | Eupalinilide B | C20H24O6 | neg | 419.1725892 | 4.166777287 | 6.99975 | M+CH_3_COO | Prenol lipids |
| 353 | 5-Hydroxy-6,7-dimethoxylflavone | C17H14O5 | neg | 343.0834348 | 3.728105119 | 6.5109 | M+HCOO | Flavonoids |
| 354 | 5-Chloro-6-methoxymellein | C11H11ClO4 | neg | 287.0349472 | 8.927142511 | 1.26745 | M+HCOO | Benzopyrans |
| 355 | Karanjin | C18H12O4 | neg | 351.0885212 | 3.979655103 | 7.547833333 | M+CH_3_COO | Flavonoids |
| 356 | Diffractic acid | C20H22O7 | neg | 409.1072831 | 3.551510098 | 7.363416667 | M+Cl | Depsides and depsidones |
| 357 | Myristic acid | C14H28O2 | pos | 292.2263036 | 4.703817449 | 8.4664 | M+CH_3_CN+Na | Fatty Acyls |
| 358 | Isoamburoside B | C21H24O10 | pos | 478.1692685 | -4.76566916 | 4.437733333 | M+NH_4_-H_2_O, M+CH_3_CN+H | Organooxygen compounds |
| 359 | Pyrrocidine B | C31H39NO4 | neg | 548.3010989 | -1.246378847 | 9.358033333 | M+CH_3_COO | Fatty Acyls |
| 360 | Alpinoid D | C20H20O3 | pos | 326.1742211 | -2.754947242 | 5.576216667 | M+NH_4_ | Diarylheptanoids |
| 361 | N-benzyl-N-methylphenylalanine | C17H19NO2 | pos | 270.1482039 | -2.420210534 | 6.132883333 | M+H | Carboxylic acids and derivatives |
| 362 | Altamisic acid | C15H20O5 | neg | 279.1246198 | 2.93613621 | 6.248416667 | M-H, 2M-H | Prenol lipids |
| 363 | Raffinose | C18H32O16 | neg | 539.1404604 | 4.014932145 | 5.045833333 | M+Cl | Organooxygen compounds |
| 364 | Gomisin L2 | C22H26O6 | neg | 491.1930899 | 2.204564945 | 5.168133333 | M+CH_3_COOH+HCOO | Tannins |
| 365 | Nudifloside C | C26H38O12 | neg | 633.2361481 | -7.116081741 | 6.787916667 | M+HCOOH+HCOO | Organooxygen compounds |
| 366 | Darutoside | C26H44O8 | neg | 529.3034646 | 3.399169392 | 11.55458333 | M+HCOO | Prenol lipids |
| 367 | beta-Glucogallin | C13H16O10 | neg | 331.068055 | 2.965517152 | 1.90945 | M-H | Tannins |
| 368 | Laciniatoside V | C27H38O14 | neg | 631.221174 | -5.427787579 | 6.446316667 | M+HCOO | Prenol lipids |
| 369 | Zedoalactone B | C15H20O5 | neg | 339.1458915 | 3.63326257 | 6.821816667 | M+CH_3_COO | Prenol lipids |
| 370 | 2'-Deoxyinosine | C10H11N4O4 | pos | 356.1191168 | -7.220894817 | 1.493283333 | M+2CH_3_CN+Na | Purine nucleosides |
| 371 | 3-Geranyl-4-methoxybenzoic acid | C18H24O3 | neg | 287.1661343 | 3.005362362 | 11.52438333 | M-H | Benzene and substituted derivatives |
| 372 | Catechin 7-arabinofuranoside | C20H22O10 | neg | 421.1154158 | 3.305490465 | 3.096433333 | M-H | Flavonoids |
| 373 | p-Hydroxy-5,6-dehydrokawain | C14H12O4 | neg | 303.0883791 | 4.180211296 | 5.757933333 | M+CH_3_COO | Kavalactones |
| 374 | 7-Hydroxy-3-(4-hydroxybenzylidene)chroman-4-one | C16H12O4 | neg | 313.0728125 | 3.930229126 | 6.5594 | M+HCOO | Homoisoflavonoids |
| 375 | 2-Hydroxyeupatolide | C15H20O4 | neg | 527.2668543 | 3.430996282 | 9.129416667 | 2M-H | Prenol lipids |
| 376 | Tanshindiol A | C18H16O5 | neg | 343.119979 | 5.784605984 | 6.592583333 | M-H+CH_3_OH | Prenol lipids |
| 377 | 4',7-Di-O-methylnaringenin | C17H16O5 | neg | 345.0991717 | 3.992215999 | 6.759 | M+HCOO | Flavonoids |
| 378 | Altholactone | C13H12O4 | neg | 263.093226 | 5.460068999 | 8.7745 | M-H+CH_3_OH | Furopyrans |
| 379 | Luteolin | C15H10O6 | neg | 285.0413659 | 3.161252991 | 7.327283333 | M-H | Flavonoids |
| 380 | Alismoxide | C15H26O2 | neg | 283.1922851 | 3.379813854 | 7.8298 | M+HCOO | Prenol lipids |
| 381 | Ligusticumic acid-methylester | C13H16O3 | pos | 262.1431606 | -5.378023456 | 11.79096667 | M+Na-H_2_O, M+CH_3_CN+H | Organooxygen compounds |
| 382 | Coleonol B | C22H34O7 | pos | 493.2928429 | 3.630892487 | 8.0064 | M+2CH_3_CN+H | Prenol lipids |
| 383 | Norlichexanthone | C14H10O5 | neg | 303.0518311 | 3.129975271 | 3.248916667 | M-H+H_2_O, M+HCOO | Benzopyrans |
| 384 | Hedragonic acid | C29H44O3 | pos | 440.3570703 | 9.595210169 | 11.22825 | M+NH_4_-H2O | Prenol lipids |
| 385 | 5-Hydroxy-7-methoxy-3-(4-hydroxybenzylidene)chroman-4-one | C17H14O5 | neg | 343.0834327 | 3.721069036 | 4.3875 | M+HCOO | Homoisoflavonoids |
| 386 | Wogonin | C16H12O5 | pos | 285.0751148 | -2.235816149 | 9.200233333 | M+H | Flavonoids |
| 387 | Rhapontigenin | C15H14O4 | neg | 303.0883561 | 3.669935134 | 7.6135 | M+HCOO | Stilbenes |
| 388 | D(+)-LACTIDE | C6H8O4 | neg | 249.0621943 | 4.412431057 | 1.4025 | M+CH_3_COOH+HCOO | Dioxanes |
| 389 | Eupatoriochromene | C13H14O3 | neg | 217.0876412 | 2.857847497 | 8.790233333 | M-H | Benzopyrans |
| 390 | Ganomycin I | C21H26O4 | neg | 447.2038748 | 4.282639155 | 8.312816667 | M+CH_3_COOH+HCOO | Prenol lipids |
| 391 | 9-Oxo-10,11-dehydroageraphorone | C15H20O2 | neg | 249.1502553 | 5.278555339 | 9.410233333 | M-H+H_2_O | Prenol lipids |
| 392 | Germacrone | C15H22O | pos | 237.1843421 | -5.148578891 | 8.506983333 | M+H2O+H | Prenol lipids |
| 393 | Tuberonic acid glucoside | C18H28O9 | neg | 405.1778143 | 4.590270546 | 3.359583333 | M-H+H_2_O | Fatty Acyls |
| 394 | toralactone | C15H12O5 | neg | 271.0620326 | 3.071270981 | 5.168133333 | M-H | Naphthopyrans |
| 395 | Ferruginol | C20H30O | pos | 328.2626786 | -4.845867451 | 13.57796667 | M+CH_3_CN+H | Prenol lipids |
| 396 | Wilforol E | C21H30O3 | pos | 372.252357 | -4.656941242 | 12.35321667 | M+CH_3_CN+H | Prenol lipids |
| 397 | 6-Methoxymellein | C11H12O4 | pos | 249.0751873 | 6.384637507 | 7.431316667 | M+H_2_O+Na | Benzopyrans |
| 398 | Kongensin A | C22H30O5 | pos | 429.2260058 | 1.821773023 | 9.3976 | M+CH_3_OH+Na | Carboxylic acids and derivatives |
| 399 | Methyl eugenol | C11H14O2 | neg | 177.0926445 | 3.03905239 | 9.48075 | M-H | Benzene and substituted derivatives |
| 400 | Norcimifugin | C15H16O6 | pos | 292.1169271 | -5.344537258 | 4.777316667 | M+NH_4_-H_2_O | Benzopyrans |
| 401 | Atractylenolide I | C15H18O2 | neg | 275.1297304 | 3.696221912 | 9.778466667 | M+HCOO | Naphthofurans |
| 402 | Hydroxyanigorufone | C19H12O3 | neg | 305.0828187 | 5.118407557 | 8.69325 | M-H+H_2_O | Naphthalenes |
| 403 | Goniodiol | C13H14O4 | neg | 279.0881944 | 3.355550434 | 6.710266667 | M+HCOO | Pyrans |
| 404 | Methyl dodonate A | C21H28O4 | neg | 389.1982795 | 3.835492776 | 9.286983333 | M+HCOO | Prenol lipids |
| 405 | 1-Stearoyl-sn-glycero-3-phosphocholine | C26H54NO7P | neg | 582.3800671 | 4.73368429 | 12.41411667 | M+CH_3_COO | Glycerophospholipids |
| 406 | Sonderianol | C20H26O2 | neg | 315.1974697 | 4.995652774 | 8.708566667 | M-H+H_2_O | Prenol lipids |
| 407 | Crucigasterin 275 | C18H29NO | pos | 294.2420238 | -4.689787466 | 12.35321667 | M+H_2_O+H | Organonitrogen compounds |
| 408 | 7,4'-Dihydroxyhomoisoflavanone | C16H14O4 | neg | 315.088478 | 3.958051021 | 6.042516667 | M+HCOO | Homoisoflavonoids |
| 409 | Maleamic acid | C4H5NO3 | neg | 132.0306143 | 8.44377697 | 15.23621667 | M-H+H_2_O, M-H+CH_3_OH | Fatty Acyls |
| 410 | Kushenol Q | C25H30O7 | neg | 459.2040751 | 5.024343809 | 9.010183333 | M-H+H_2_O | Flavonoids |
| 411 | Coriandrin | C13H10O4 | neg | 247.0618898 | 5.568336726 | 7.42655 | M-H+H_2_O | Isocoumarins and derivatives |
| 412 | Costunolide | C15H20O2 | pos | 250.1796692 | -2.094225265 | 9.9557 | M+NH_4_ | Prenol lipids |
| 413 | Epicatechin | C15H14O6 | neg | 307.0831537 | 4.879790813 | 3.959583333 | M-H+H_2_O | Flavonoids |
| 414 | N-Acetyl-DL-tryptophan | C13H13N2O3 | neg | 290.0890634 | -7.156211347 | 15.22118333 | M+HCOO | Carboxylic acids and derivatives |
| 415 | Muurol-4-ene-3,8-dione | C15H22O2 | neg | 265.1816868 | 5.581022832 | 8.85895 | M-H+CH_3_OH | Prenol lipids |
| 416 | Attenuol | C19H20O3 | pos | 261.1302542 | 9.666465455 | 14.8882 | M+H-2H_2_O | Aryltetralin lignans |
| 417 | Liquiritin | C21H22O9 | neg | 417.121104 | 4.778819192 | 15.2492 | M-H | Flavonoids |
| 418 | Desoxo-narchinol A | C12H16O2 | pos | 233.1167629 | 7.460004156 | 9.709483333 | M+H_2_O+Na | Organooxygen compounds |
| 419 | Perillene | C10H14O | pos | 150.127453 | -5.410127758 | 7.111466667 | M+NH_4_-H_2_O | Heteroaromatic compounds |
| 420 | Epi-Cryptoacetalide | C18H22O3 | neg | 303.1610843 | 5.206173245 | 9.062533333 | M-H+H_2_O | Naphthofurans |
| 421 | Atractylodin | C13H10O | pos | 201.0906181 | -5.20023634 | 10.40556667 | M+H_2_O+H | Heteroaromatic compounds |
| 422 | Erucic acid | C22H42O2 | neg | 355.3228094 | 4.814337348 | 13.55083333 | M-H+H_2_O | Fatty Acyls |
| 423 | Incensole | C20H34O2 | pos | 306.27868 | -3.266453471 | 13.34695 | M+NH_4_-H_2_O | Prenol lipids |
| 424 | 7-Dehydrobrefeldin A | C16H22O4 | neg | 277.1452395 | 2.540758456 | 8.24405 | M-H | Macrolides and analogues |
| 425 | 8-Acetoxypentadeca-1,9Z-diene-4,6-diyn-3-ol | C17H22O3 | neg | 319.1561534 | 3.862072358 | 8.414616667 | M+HCOO | Fatty Acyls |
| 426 | Neoprocurcumenol | C15H22O2 | neg | 279.1609407 | 3.248888247 | 8.330033333 | M+HCOO | Prenol lipids |
| 427 | Chrysosplenetin B | C19H18O8 | neg | 373.0940161 | 3.007106413 | 9.286983333 | M-H | Flavonoids |
| 428 | Atractylenolide III | C15H20O3 | pos | 249.1479441 | -2.324496257 | 10.57093333 | M+H-2H_2_O, M+H | Prenol lipids |
| 429 | 3-Clerodene-2,15-diol, 15-Carboxylic acid, 2-keton | C20H32O3 | neg | 337.2395206 | 5.23276969 | 11.1424 | M-H+H_2_O | Prenol lipids |
| 430 | 2-Adamantanone | C10H14O | neg | 195.1032713 | 4.038885033 | 9.98465 | M+HCOO | Organooxygen compounds |
| 431 | Bipolal | C24H30O5 | neg | 443.2089457 | 3.570129747 | 15.13228333 | M+HCOO | Prenol lipids |
| 432 | 4-Hydroxy-11,12,13-trinor-5-eudesmen-7-one | C12H18O2 | neg | 239.1296429 | 3.930614681 | 8.414616667 | M+HCOO | Organooxygen compounds |
| 433 | Perilloxin | C16H18O4 | neg | 273.1141158 | 3.221943049 | 8.2617 | M-H | Benzoxepines |
| 434 | Ginkgolic acid (C13:0) | C20H32O3 | neg | 319.2287033 | 2.607051796 | 12.9542 | M-H | Benzene and substituted derivatives |
| 435 | Linamarin | C10H17NO6 | neg | 264.1095717 | 5.197908033 | 0.82145 | M-H+H_2_O | Organooxygen compounds |
| 436 | L-LYSINE | C6H14N2O2 | pos | 147.1124896 | -2.152947575 | 0.7214 | M+H | Carboxylic acids and derivatives |
| 437 | Dihydrocoriandrin | C13H12O4 | neg | 231.0666747 | 1.690084186 | 6.57645 | M-H | Benzopyrans |
| 438 | 5-Hydroxy-2-pyrrolidinone | C4H7NO2 | neg | 136.0169392 | -1.391193308 | 4.6858 | M+Cl | Pyrrolidines |
| 439 | Clausine E | C14H10NO3 | neg | 239.0603373 | 6.437936485 | 14.32095 | M-H | Indoles and derivatives |
